# Shh and Olig2 sequentially regulate oligodendrocyte differentiation from hiPSCs for the treatment of ischemic stroke

**DOI:** 10.7150/thno.69217

**Published:** 2022-03-28

**Authors:** Jian Xu, Jingxin Zhao, Rui Wang, Yidi Zhang, Lan Shen, Qian Xiao, Yuan Xie, Jinjun Jiang, Yichu Nie, Wenbin Deng

**Affiliations:** 1 School of Pharmaceutical Sciences (Shenzhen), Sun Yat-sen University, Shenzhen, 510631, China.; 2 Clinical Research Institute, First People's Hospital of Foshan, Foshan, 528000, China.; 3 Department of Pathology, Shenzhen Hospital of Southern Medical University, Shenzhen, 518000, China.; 4 Department of Biochemistry and Molecular Medicine, University of California, Davis, Sacramento, CA, USA.

**Keywords:** Olig2, Sonic hedgehog (Shh), Oligodendrocyte progenitor cells (OPCs), Human induced pluripotent stem cells (hiPSCs), Ischemic stroke.

## Abstract

**Rationale**: Demyelination is a major component of white matter injury, characterized by oligodendrocyte (OL) death and myelin sheath loss, which result in memory loss and cognitive impairment in the context of ischemic stroke. Accumulating evidence has shown that OLs can be generated by the direct activation of defined transcription factors (TFs) in human induced pluripotent stem cells (hiPSCs); however, the rapid acquisition of single TF-induced OL progenitor cells (OPCs) as cell therapy for ischemic stroke remains to be thoroughly explored.

**Methods**: A stable, chemically defined protocol was used to generate a substantial number of transplantable and functional OLs through the partial inhibition of sonic hedgehog (Shh) activity by GANT61 during neural induction from hiPSCs and sequential induction of TF Olig2 overexpression. Transcriptome and metabolome analyses further revealed a novel molecular event in which Olig2 regulates OL differentiation from hiPSC-derived neural progenitor cells (NPCs). Olig2-induced NG2^+^ OPCs (Olig2-OPCs) were then evaluated for their therapeutic potential in cell-based therapy for ischemic stroke.

**Results:** GANT61 treatment resulted in a motor neuron (MN)-OL fate switch during neural induction, and induced overexpression of Olig2 accelerated oligodendroglial lineage cell specification. Olig2-OPCs expressed typical oligodendroglial lineage marker genes, including *NKX2.2*, *CSPG4*, and *ST8SIA1*, and displayed superior ability to differentiate into mature OLs *in vitro*. Mechanistically, Olig2-OPCs showed increased gene expression of the peroxisome proliferator-activated receptor γ (PPARγ) signaling pathway, and activated CEPT1-mediated phospholipogenesis. Functionally, inhibiting PPARγ and knocking down *CEPT1* further compromised the terminal differentiation of Olig2-OPCs. Most importantly, when transplanted into a rat model of transient middle cerebral artery occlusion (tMCAO), Olig2-OPCs efficiently promoted neurological functional recovery by reducing neuronal death, promoting remyelination, and rescuing spatial memory decline.

**Conclusions:** We developed a stable, chemically defined protocol to generate OPCs/OLs with partial inhibition of Shh activity by GANT61 from hiPSCs and sequentially induced the expression of the single TF Olig2. Olig2-OPC transplantation may be an ideal alternative approach for ischemic stroke rehabilitation therapy.

## Introduction

Ischemic stroke is a progressive neurodegenerative syndrome characterized by loss of the myelin sheath and death of oligodendrocytes (OLs), which are generated from OL progenitor cells (OPCs) via a consecutive process of cell cycle exit, maturation, and differentiation 1. The disintegrated myelin sheath further results in long-term memory loss and cognitive impairment in ischemic stroke patients 2-4. In recent years, stem cell therapy has become a research hotspot in the field of cell replacement therapy for ischemic stroke 5, 6. Although the main types of transplanted cells in clinical studies are neural stem cells (NSCs)/neural progenitor cells (NPCs), it is believed that implanted NSCs/NPCs mainly differentiate into neurons and astrocytes *in vivo*, and few differentiate into OLs 7-9. Indeed, the development of new therapeutic strategies for the repair of myelin injury may be more effective in promoting functional recovery from ischemic stroke. The use of exogenous OPCs derived from human induced pluripotent stem cells (hiPSCs) is currently recognized as a potential method for improving myelin sheath repair. In a previous study, hiPSC-derived OPC transplantation resulted in differentiation and the formation of functional myelin sheaths in the brains of transgenic mice with loss of myelination 10. However, the procedures for rapidly and stably obtaining many transplantable human OPCs *in vitro* are highly complex, which restricts the further clinical application of human OPC transplantation.

Recently, a promising approach to improve the generation efficiency of OLs by transfecting cells with viral vectors overexpressing OPC/OL fate-related transcription factors (TFs), such as *SOX10,* and indirectly inducing hiPSC-derived NPC differentiation into OPCs has been reported 11, 12. However, as *SOX10* is known to be involved in OL terminal differentiation, overexpression of *SOX10* only induces late OPC/OL specification. Very few neural/glial antigen 2 (NG2)- or platelet-derived growth factor α (PDGFRα)-positive early OPCs were found in *SOX10*-induced cultures, suggesting that *SOX10* induces only the direct generation of late OPCs without an intermediate OPC stage 12. Studies have proven that NG2^+^ OPCs not only rapidly proliferate and migrate to the ischemic region under ischemia but also differentiate into OLs to form functional myelin, promoting recovery from injury, which is widely regarded as a promising strategy for the treatment of ischemic stroke 13. Hence, another TF should be considered to directly drive NG2^+^ OPC generation. Olig2 is one of the core TFs first identified for the development of OLs in the human central nervous system (CNS) and plays an important role in the differentiation and maturation of OPCs and myelination 14. Additionally, Olig2 is believed to orchestrate NG2^+^ OPC differentiation by inducing the expression of the downstream factors *NKX2.2* and *SOX10* in mouse and human embryonic stem cells (ESCs) 15. However, direct activation of Olig2 cannot achieve rapid differentiation of OPCs derived from hiPSCs *in vitro*, or its effect has been considered weak 16, 17. In fact, Olig2 plays sequential dual roles in promoting motor neurons (MNs) at early neurogenesis, promoting OLs and repressing astrocytes at the later gliogenesis stage 18. Therefore, we speculated that the overexpression of Olig2 in NPCs may promote MN production at an early stage, causing the failure of OL generation. Thus, inhibiting the differentiation of MNs in the early stage of neural induction may promote OPC generation. Sonic hedgehog (Shh) activity is necessary for the pattern of the pMN domain and MN formation 19. Notably, there is a temporal partial downregulation of Shh signaling downstream of GLI1 in NSCs residing in the CNS prior to their differentiation into OLs and mobilization to the demyelination site in a cuprizone-induced demyelination mouse model 20. This finding suggests that temporal partial inhibition of the Shh regulatory pathway is required for OL differentiation and maturation from NPCs. Here, we used GANT61 (a Shh signaling inhibitor) in our modified protocols to inhibit MN production during neural induction from hiPSCs and subsequently obtained comparable OPCs (Olig2-OPCs) by ectopic expression of Olig2.

Peroxisome proliferator-activated receptor γ (PPARγ) is a ligand-activated TF that is widely believed to control the proliferation, differentiation and lipid metabolism of neurons and glial cells in the CNS 21-23. Roth et al*.* further demonstrated that PPARγ has a role in the differentiation and function of OLs, and these effects were blocked by the PPARγ antagonist GW9662 24. Given that PPARγ expression plays an important role in the differentiation of NPCs to OPCs, whether Olig2 expression affects PPARγ expression and cellular metabolic remodeling and whether this metabolic remodeling determines the fate of OPCs remain unclear. In this study, the transcriptional and metabolic profiles of Olig2-OPCs were analyzed to explore the regulatory mechanism by which Olig2 affects the differentiation of OPCs. Additionally, Olig2-OPC transplantation was further used to evaluate the efficacy against ischemic stroke.

## Results

### Transient Shh inhibition by GANT61 and Olig2 overexpression sequentially promoted NG2^+^ OPC differentiation from hiPSCs

The tetracycline-inducible expression system was first established in hiPSCs 18 (Figure S1A, referred to Olig2/hiPSCs). Olig2 was overexpressed by doxycycline treatment (1 μg/mL, Figure S1B-C). To identify which conditions possessed the potential of OL differentiation, hiPSCs were maintained for 14 days along with neural induction and oligodendroglial induction and then subjected to total RNA extraction for quantitative polymerase chain reaction (qPCR) analysis of *SOX10* mRNA expression, a highly specific marker of late-stage OPCs, at d14 (Figure S2A). In line with previous results, Olig2 overexpression alone did not induce *SOX10* mRNA expression. GANT61 treatment (5 μM) alone had a weaker effect on *SOX10* mRNA; however, only GANT61 combined with Olig2 was capable of inducing significant upregulation of *SOX10* mRNA expression (Figure S2B).

**Figure 1:**
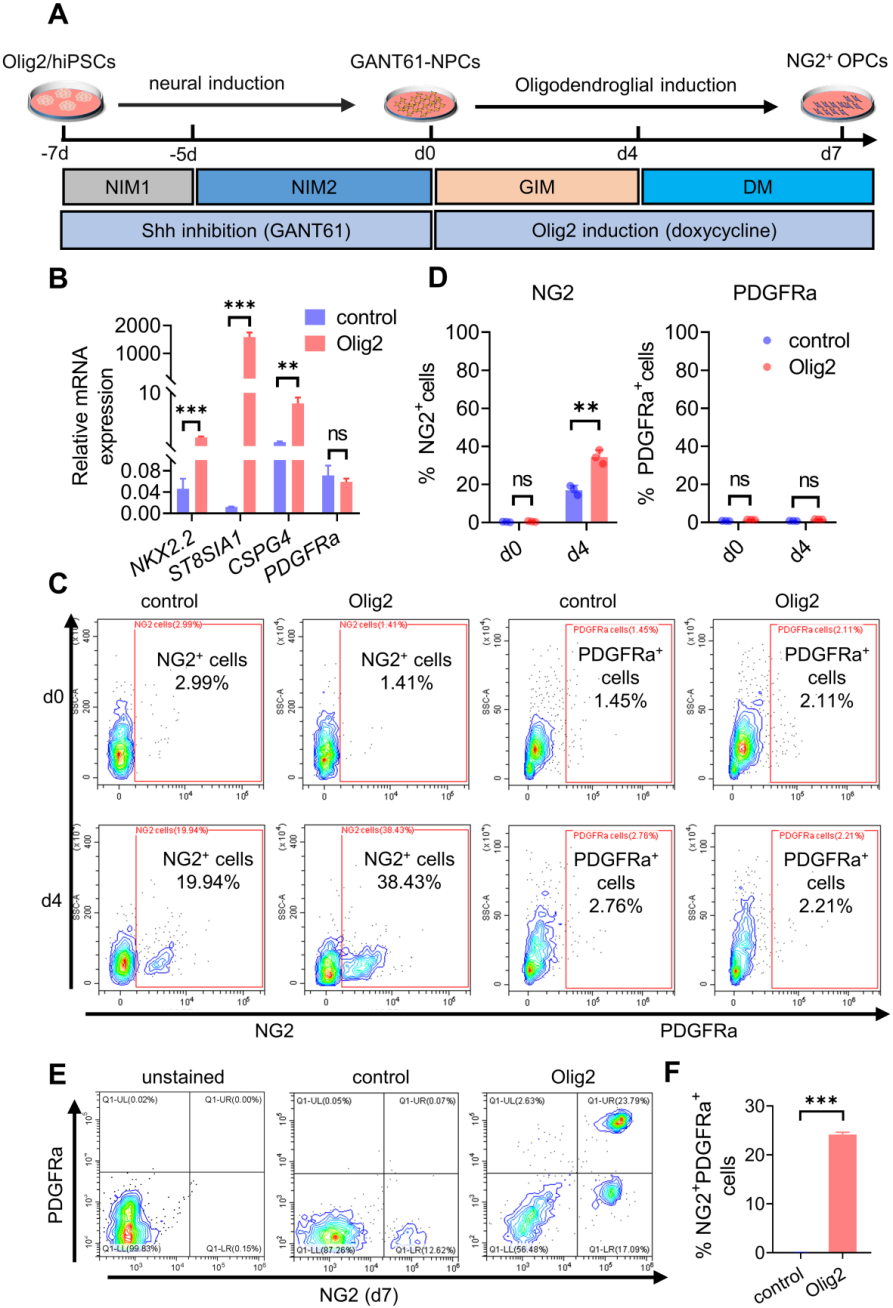
** Transient Shh inhibition by GANT61 and Olig2 overexpression sequentially promoted NG2^+^ OPC differentiation from hiPSCs. A** Strategy for generating OPCs derived from Olig2/hiPSCs treated with GANT61 during neural induction and sequentially inducing Olig2 overexpression by doxycycline treatment. Adherent hiPSC colonies were differentiated into NPCs (GANT61-NPCs) by adding GANT61 to NIM1 and NIM2 from d-7 to d0. The cultured GANT61- NPCs were dissociated into single cells in six-well plates precoated with poly-L-ornithine/laminin and then subjected to glial induction with GIM and DM containing Y-27632. **B** The mRNA expression levels of oligodendroglial lineage genes (*NKX2.2*, *CSPG4, ST8SIA1,* and *PDGFRa)* involved in OL development at d4 after Olig2 induction (** p < 0.01, *** p < 0.001, by a two-tailed Student's *t* test). **C** Representative flow cytometry analyses for the expression of NG2 and PDGFRα in the control and Olig2 induction cultures at d4 after Olig2 induction. **D** The corresponding quantification of NG2^+^ and PDGFRα^+^ cells at d4 in control and Olig2-induced cultures (ns p > 0.05, ** p < 0.01, by a two-tailed Student's *t* test). Representative flow cytometry analyses (**E**) and quantification (**F**) of the coexpression of NG2 and PDGFRα in the control and Olig2-induced cultures at d7 of differentiation after Olig2 induction (*** p < 0.001, by a two-tailed Student's *t* test). The graphs represent the individual data points and the mean ± SEM of three independent experiments. Immunofluorescence images are representative of n = 3 biological replicates.

Herein, we developed a scheme to generate OPCs from Olig2/hiPSCs with transient and selective partial inhibition of Shh activity by GANT61 during neural induction, followed by induction of Olig2 overexpression. The GANT61-NPCs generated from Olig2/hiPSCs (Figure S3A-C) were cultured in glial induction medium (GIM) with doxycycline for 4 days, and the medium was subsequently replaced with differentiation medium (DM, Figure 1A). qPCR results showed that forced expression of Olig2 significantly upregulated the transcription of oligodendroglial lineage (*NKX2.2*) and surface protein marker genes (*CSPG4*, and *ST8SIA1*); however, there was no significant change in *PDGFRa* (Figure 1B) at d4. Because *ST8SIA1* is not a strictly oligodendroglial lineage gene, *PSA-NCAM*^+^, a joint marker, is usually required to identify OPCs 25. Therefore, we focused on the production of NG2^+^ cells induced by Olig2 overexpression. To further confirm the generation of NG2^+^ cells, we analyzed the cultured cells at d7 by flow cytometry. The Olig2-induced cultures largely comprised NG2^+^ cells (referred to as Olig2-OPCs, 38.43 ± 3.67%) compared to the control-NG2^+^ OPCs (referred to as control-OPCs, 19.94 ± 2.47%); however, PDGFRa^+^ cells were still largely absent at this time point (Figure 1C-D). These results suggested that these NG2^+^ cells, unlike mature OPCs, could be early oligodendroglial lineage cells.

We further demonstrated that the GANT61-NPCs were converted to NG2^+^/Olig2^+^ cells after culture in DM. Many SOX10- and A2B5-positive cells were observed, but PDGFRa^+^ cells were still not observed at d4 (Figure S3D). However, after promotion of OL terminal differentiation for 3 days, some fraction of early NG2^+^ cell-derived differentiated cells coexpressed NG2 and PDGFRa proteins (Figure 1E-F, Figure S3E), implying that some early NG2^+^ cells converted to mature OPCs. Taken together, these results suggested that Shh inhibition and Olig2 overexpression promoted OPC differentiation from hiPSCs.

### Transient Shh inhibition abolished MN development and facilitated the MN-OL fate switch by inhibiting Olig2 phosphorylation at Ser147

**Figure 2:**
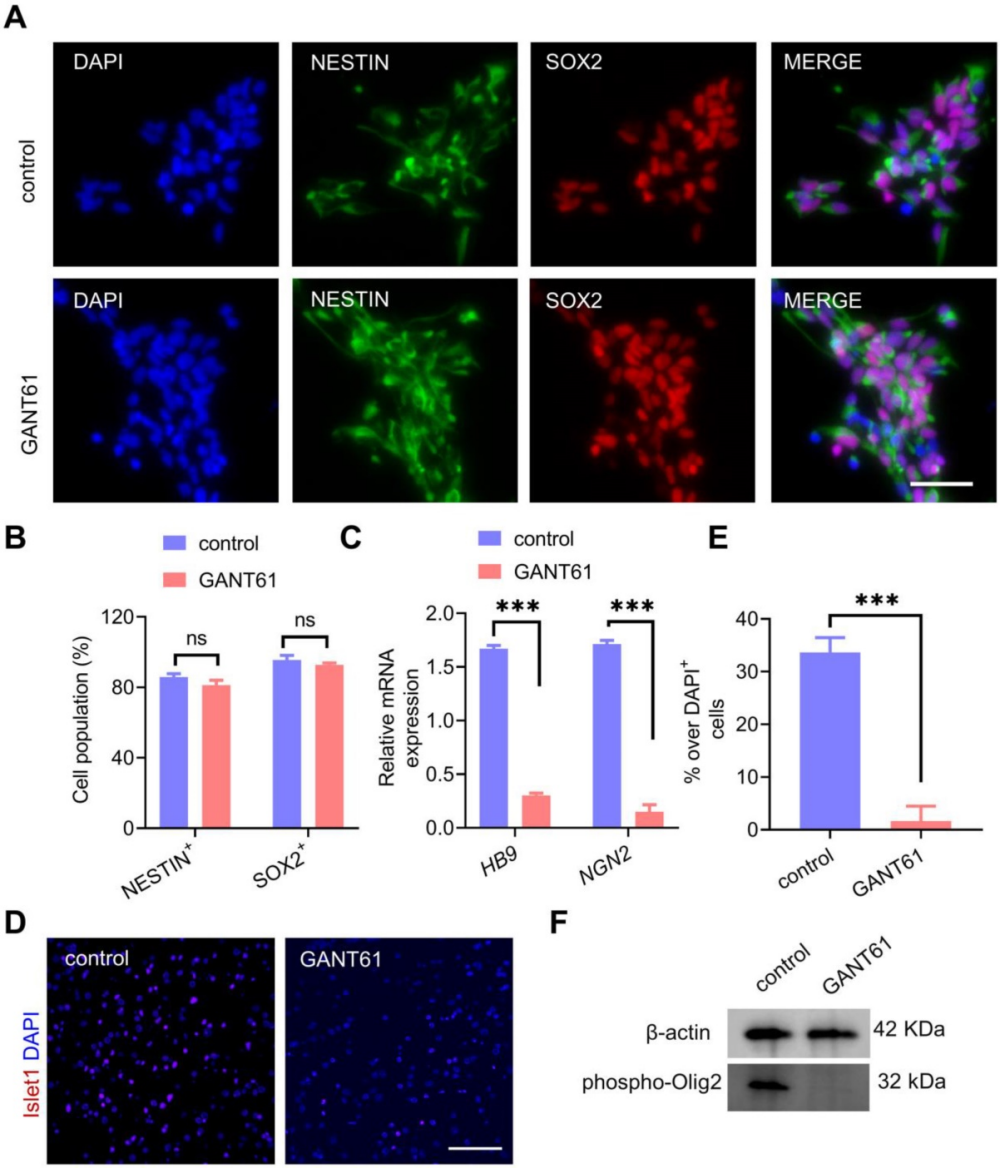
** Shh inhibition abolished MN formation and facilitated the MN-OL fate switch by blocking Olig2 phosphorylation at Ser147. A** Representative immunofluorescent staining images of control NPCs and GANT61-NPCs showed coexpression of NPC markers (NESTIN and PAX6) at d0 of differentiation; scale bar, 50 μm. **B** The corresponding quantification of NESTIN^+^/PAX6^+^ NPCs from control NPCs and GANT61-NPCs (at d0 of differentiation, ns p > 0.05, a two-tailed Student's t test). GANT61 treatment did not affect NPC formation from hiPSCs. **C** The mRNA expression levels of MN marker genes (*HB9* and *NGN2*) were analyzed by qPCR in control NPCs and GANT61 NPCs (at d0 of differentiation, *** p < 0.001, by a two-tailed Student's t test). GANT61 treatment downregulated MN-specific marker genes (*HB9* and *NGN2*). **D** Representative immunofluorescent staining images of the MN marker Islet1 in control NPCs and GANT61-NPCs; scale bar, 100 μm. **E** Quantification of the expression of Islet1 in the control NPCs and GANT61 NPCs. GANT61 treatment downregulated the MN-specific protein marker Islet1 (*** p < 0.001, by a two-tailed Student's t test). **F** Western blot analysis of Olig2 phosphorylation at Ser147. GANT61 treatment blocked Olig2 phosphorylation at Ser147. Control-NPCs and GANT61-NPCs were immunoblotted with an anti-Olig2 (phospho-S147) antibody, and proteins were normalized to the housekeeping gene β-actin. The graphs represent the individual data points and the mean ± SEM of three independent experiments. Immunofluorescence images are representative of n = 3 biological replicates.

In an effort to determine the underlying mechanisms of partial Shh inhibition during neural induction on OPC formation induced by ectopic overexpression of Olig2, we first sought to investigate whether transient and partial Shh inhibition affected NPC formation from hiPSCs. The generated NPCs were characterized in the presence of GANT61 or vehicle treatment alone. We found that both control-NPCs and GANT61-NPCs differentiated into NESTIN^+^/SOX2^+^ cells with similar populations (Figure 2A-B). This result suggested that Shh signaling inhibition did not affect hiPSC differentiation into NPCs. Moreover, qPCR analysis further showed that the mRNA expression levels of the neural progenitor-specific markers *PAX6* and *NESTIN* in GANT61-NPCs were highly upregulated, whereas the expression of the endogenous pluripotency markers *OCT4* (*POU5F1*) and *NANOG* decreased rapidly during the neural differentiation of hiPSCs (Figure S4A). Interestingly, qPCR results showed that the levels of the MN-specific TFs *NGN2* and *HB9* were significantly decreased in GANT61-NPCs (Figure 2C); however, the levels of the oligodendroglial-specific lineage marker genes *SOX10*, *NKX2.2* and *SOX9* remained unchanged (Figure S4B). Accordingly, Islet1 is required for the formation of stem cell-derived MNs 26. Our immunostaining assays showed that sustained culture for MN differentiation in GANT61-NPCs resulted in a lower proportion (close to 0) of Islet1^+^ cells (Figure 2D-E). These results indicated that temporal and partial inhibition of Shh signaling during neural induction from hiPSCs could block MN generation without affecting differentiation toward oligodendrogenesis. Since mutating Ser147 in Olig2 could abolish MN production 27, we next measured the phosphorylated Olig2 level in GANT61-NPCs and found that Ser147 phosphorylation of Olig2 was essentially blocked in GANT61-NPCs (Figure 2F). Taken together, these results revealed that GANT61 blocked Olig2 phosphorylation at Ser147 and downregulated *NGN2* expression, suggesting that GANT61 inhibited MN production and might have triggered the MN-OL fate switch.

### Maturation of Olig2-OPCs into OLs

**Figure 3:**
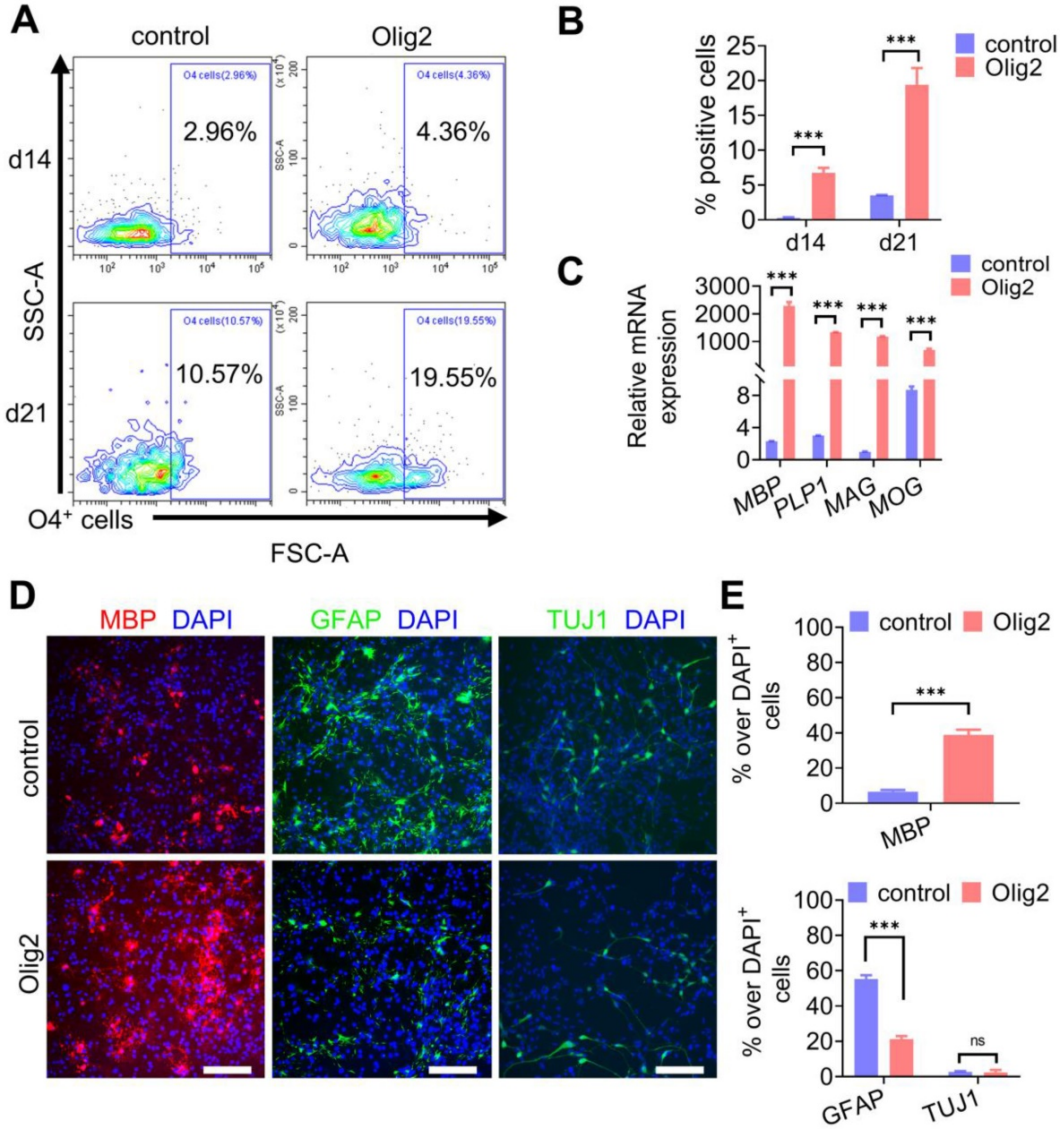
** Maturation of Olig2-OPCs into OLs. A** Representative flow cytometry analyses for the expression of O4^+^ cells at d7, d14 and d21 of differentiation. **B** Quantification of O4^+^ cells in control and Olig2 induction cultures at d7, d14 and d21 of differentiation (*** p < 0.001, by a two-tailed Student's t test). **C** The mRNA expression levels of* MBP*, *PLP1, MAG*, and *MOG* at d21 of OL differentiation (*** p < 0.001, by a two-tailed Student's t test). **D** Immunostaining of MBP, GFAP and TUJ1 at d21 of differentiation; scale bars, 100 μm. **E** Quantification of MBP^+^ OLs, GFAP^+^ astrocytes and TUJ1^+^ neurons in the control and Olig2 induction cultures at d21 of differentiation (ns p > 0.05, *** p < 0.001, by a two-tailed Student's t test). The graphs represent the individual data points and the mean ± SEM of three independent experiments. Immunofluorescence images are representative of n = 3 biological replicates.

Next, we questioned whether Olig2-OPCs could mature into OLs. Sustained cultures were maintained in DM, and the cultured cells started to present a branched morphology and coexpress the O4 epitope and the more mature OL marker MBP (Figure S5A-B). To determine the kinetics, efficiency and yield of Olig2-mediated oligodendroglial lineage specification, we performed weekly flow cytometric analyses of O4^+^ OL generation in our cultures. Flow cytometry analysis indicated that Olig2 induced O4^+^ OLs, starting from 4.36 ± 3.0% O4^+^ cells at d7 to 19.55 ± 11.1% O4^+^ cells by d21; however, in contrast, only 10.57 ± 0.5% O4^+^ cells were identified in the control-transduced cell cultures at d21 (Figure 3A-B). Additionally, starting NPCs (GANT61-NPCs) were plated at 500,000 cells/well on poly-L-ornithine/laminin-coated six-well plates. The cultured cells were counted to calculate the number of O4^+^ OLs at d14 and d21 of differentiation by flow cytometry. The yields of O4^+^ OLs ranged from 35% ± 6.61 at d14 to 119% ± 19% at d21 after Olig2 overexpression (Figure S5C). Very low yields of O4^+^ mature OLs from the control group were obtained using this protocol because most of the cells died gradually over more than 2 weeks. These data suggested an expansion of Olig2-expressing cells during our differentiation protocol. At d21 of differentiation, the Olig2-induced cultures showed 989-fold upregulated levels of *MBP* compared to the control cultures. This upregulation was also associated with a concurrent increase in other myelin genes, i.e., myelin-associated glycoprotein (*MAG*) by 1200-fold, myelin OL glycoprotein (*MOG*) by 90-fold, and proteolipid 1 (*PLP1*) by 447-fold (n = 3, Figure 3C). In line with qPCR results, immunofluorescence staining for oligodendroglial markers (MBP) further confirmed that Olig2 overexpression led to the rapid differentiation and maturation of GANT61-NPCs (Figure 3D-E). Furthermore, few GFAP^+^ cells were observed in the Olig2-induced cultures at d21 of differentiation, which indicated a low potential for differentiation into astrocytes (Figure 3D-E). Because GANT61 treatment inhibited the production of neurons, pretreatment with GANT61 during neuronal induction greatly reduced the production of neurons. In this case, very few neurons were generated in both the control and Olig2-overexpression groups. These data demonstrated that Olig2 overexpression could eventually facilitate OPC differentiation and maturation into OLs and inhibit astrocyte generation *in vitro*.

### Olig2 overexpression altered the expression of genes and metabolites involved in PPARγ-mediated phospholipid biosynthesis

**Figure 4:**
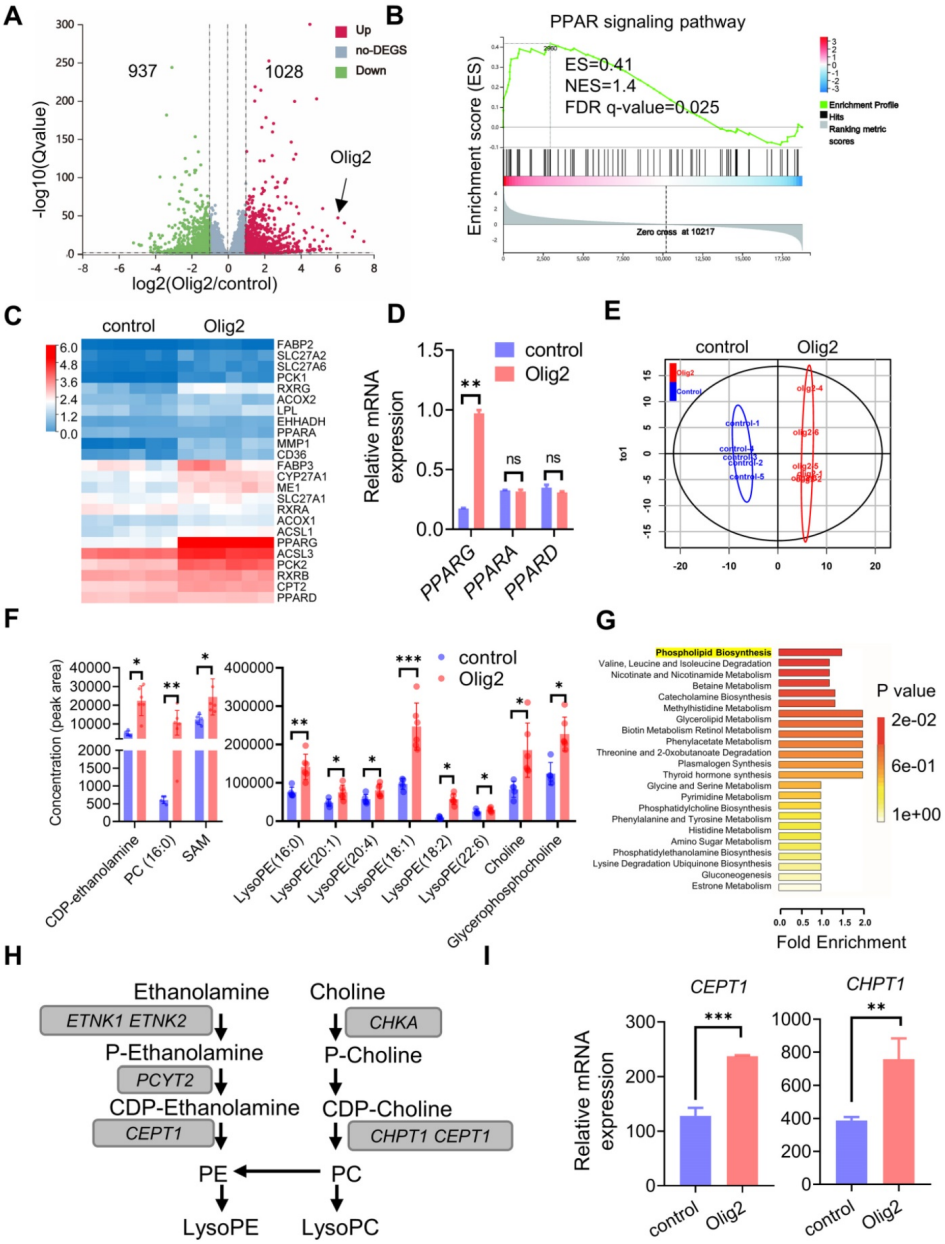
** Olig2 overexpression altered the expression of genes and metabolites involved in the PPARγ-mediated phospholipogenesis pathway. A** Volcano plots of significantly differentially expressed genes (DEGs). Transcriptome analysis of Olig2-OPCs and control-OPCs (n = 6), red, upregulated; green, downregulated. **B** Gene set enrichment analysis (GSEA) indicated strong enrichment of the PPAR signaling pathway in Olig2-OPCs. **C** Heatmap evaluation of the expression of genes linked to lipid metabolism. **D** The mRNA expression levels of *PPARG*, *PPARA*, and *PPARD* in Olig2-OPCs and control-OPCs (ns p > 0.05, ** p < 0.01, by a two-tailed Student's t test). **E** PCA score plots of HILICESI+-MS metabolomics profiles obtained from HILIC-ESI+-MS, control-OPCs, n = 5; Olig2-OPCs, n = 6. **F** Metabonomic analysis showed that several species of intermediates and hydrolysates of phospholipid biosynthesis were increased in Olig2-OPCs (n = 5/6, * p < 0.05, ** p < 0.01, *** p < 0.001, by a two-tailed Student's t test). **G** Metabolite set enrichment analysis (MSEA) showed differential metabolites highly enriched in phospholipid biosynthesis in Olig2-OPCs. **H** The mRNA expression levels of *CEPT1* and *CHPT1* in Olig2-OPCs and control-OPCs (** p < 0.01, *** p < 0.001, by a two-tailed Student's t test). The graphs represent the individual data points and the mean ± SEM of three independent experiments.

To further determine the detailed mechanism of the effect of Olig2 overexpression on OPC differentiation and maturation, we performed RNA-seq analysis of purified Olig2-OPCs and control-OPCs at d7 of differentiation. Analysis of the RNA-seq read distribution across specific genes confirmed the overexpression of Olig2 in Olig2-OPCs (Figure 4A). Differentially expressed gene (DEG) analysis revealed that Olig2 overexpression upregulated the expression of 1028 genes and repressed the expression of 937 genes in Olig2-OPCs (Figure 4A). Gene set enrichment analysis (GSEA) showed that the genes with upregulated expression were also enriched for positive regulators of PPAR signaling (Figure 4B). Consistent with the GSEA pathway enrichment analysis, KEGG pathway enrichment analysis identified a strongly positive association of upregulated expression with the PPAR signaling pathway (Figure S6A). These data demonstrated that Olig2-OPCs altered the expression of genes involved in the PPAR signaling pathway. Next, we further analyzed the major DEGs involved in the PPAR signaling pathway and found that Olig2-OPCs strongly activated the expression of PPAR and its ligand, *RXG,* and even target genes, including *FABP3, ME1, CD36, CYP27A1* and *CPT2* (Figure 4C). qPCR results further confirmed the upregulation of PPARγ/*PPARG* expression, while* PPARA* and *PPARD* did not show significant changes in Olig2-OPCs (Figure 4D). These results suggested that PPARγ could be the dominant TF in the activated PPAR signaling pathway. Previous studies have shown that PPARγ activation affects free acid uptake and storage, and lipogenesis 28, which indicates that the PPARγ-mediated PPAR signaling pathway is highly related to cell metabolism. Thus, we further examined the metabolic process of Olig2-OPCs through nontargeted metabolomics. Nonregulatory principal component analysis (PCA) showed a clear distinction between the two groups of cell samples, suggesting that these two groups of OPC metabolic profiles showed significant differences (Figure 4E). We then found that the main metabolites and derivatives of phospholipid synthesis, such as the hydrolytic products of phosphatidylcholine (PC), phosphatidylethanolamine (PE), choline, and CDP-ethanolamine, which are controlled through the upregulation of* CEPT1* and *CHPT1* expression, were significantly increased in Olig2-OPCs (Figure 4F). More importantly, metabolite set enrichment analysis further identified activated phospholipid synthesis pathways based on the differentially abundant metabolites (Figure 4G). The higher mRNA expression levels of *CEPT1* and *CHPT1* in Olig2-OPCs, key rate limiting enzymes in the synthesis of PC and PE, were further validated by qPCR (Figure 4H-I). Thus, Olig2-OPCs showed higher phospholipogenesis activity that was, at least in part, mediated by PPARγ expression.

### Olig2 interacted with PPARγ and participated in phospholipogenesis in OL differentiation partially through SMARCA4 (Brg1) expression

**Figure 5:**
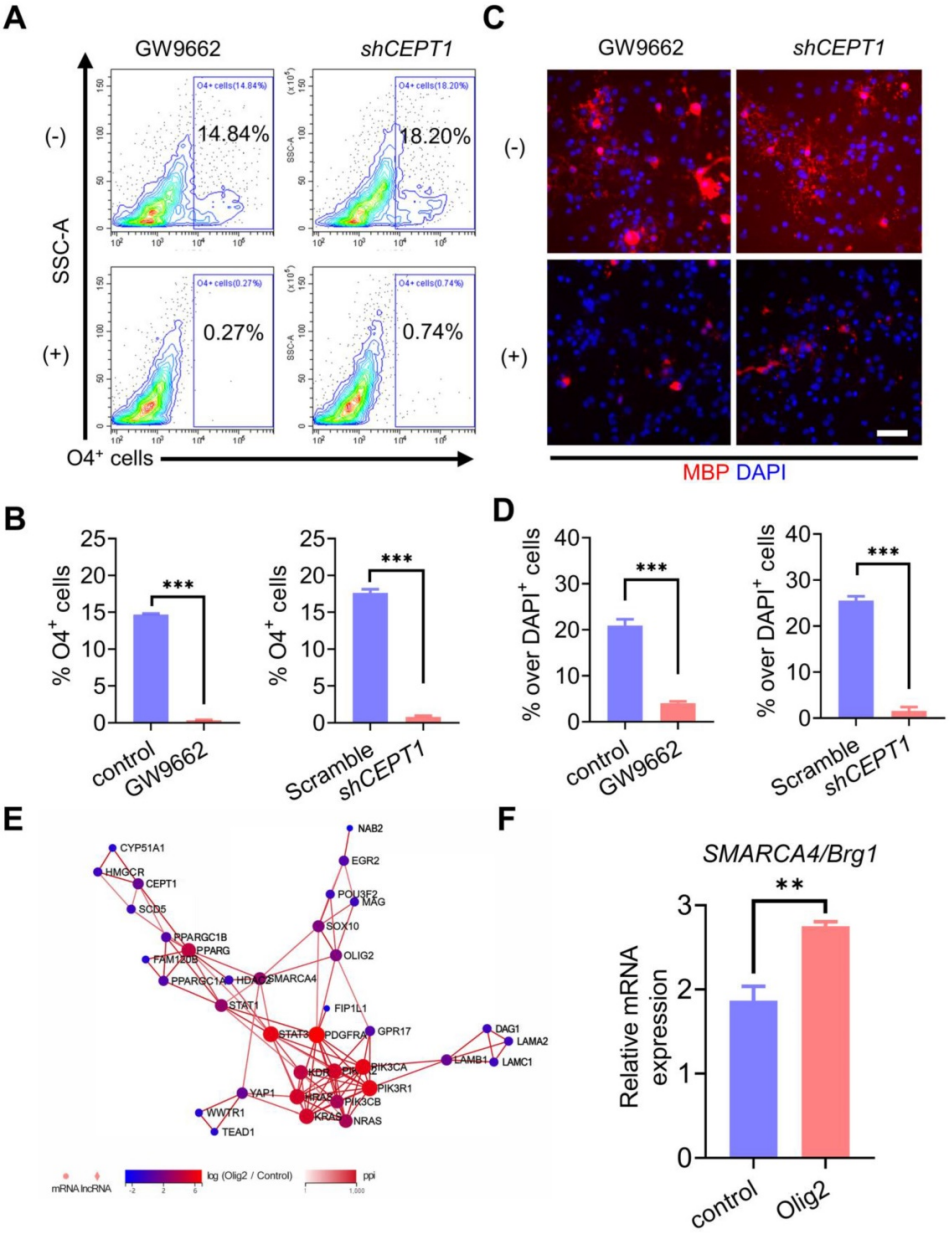
** Olig2 interacted with PPARγ and participated in phospholipogenesis involved in OL differentiation partially through SMARCA4 (Brg1) expression. A** Flow cytometric analysis of the effect of pharmacological inhibition of PPARγ or *CEPT1* knockdown on Olig2-OPC differentiation, and O4^+^ cells were determined at d21 of differentiation. **B** Quantification of O4^+^ cells at d21 of differentiation (*** p < 0.001, by a two-tailed Student's t test). **C** Immunostaining analysis of MBP expression to explore the effect of pharmacological inhibition of PPARγ or CEPT1 knockdown on Olig2-OPC differentiation at d21 of differentiation (scale bar, 50 μm). **D** Quantification of MBP^+^ cells at d21 of differentiation (*** p < 0.001, by a two-tailed Student's t test). **E** PPI networks among the main differentially expressed genes in control-OPCs and Olig2-OPCs indicated that overexpression of Olig2 might promote OL differentiation partially through the SMARCA4/Brg1-PPARG-CEPT1 signaling axis. **F** qPCR results indicated that the mRNA expression level of *SAMRCA4/Brg1* was significantly upregulated in Olig2-OPCs (** p < 0.01, by a two-tailed Student's t test). The graphs represent the individual data points and the mean ± SEM of three independent experiments. Immunofluorescence images are representative of n = 3 biological replicates.

To clarity the underlying mechanism by which Olig2 overexpression could promote oligodendrogenesis in PPARγ-mediated phospholipogenesis, we employed both pharmacological and genetic approaches to inhibit PPARγ expression or phospholipid biosynthesis. For the pharmacological inhibition of PPARγ, we treated Olig2-OPCs with the well-characterized PPARγ antagonist GW9662 along with OL induction at d7 of differentiation. For the genetic inhibition of phospholipid biosynthesis, *CEPT1*, a rate-limiting enzyme involved in phospholipogenesis was knocked down in Olig2-OPCs (Figure S7A-B). The percentage of generated O4^+^ cells was analyzed by flow cytometry at d21 of differentiation. We observed markedly decreased O4^+^ cells in Olig2-OPCs with GW9662 treatment or *CEPT1* knockdown (Figure 5A-B). Furthermore, similar to the findings in O4^+^ cells, MBP staining also showed significantly reduced mature MBP^+^ OLs following GW9662 treatment or *CEPT1* knockdown at d21 of differentiation (Figure 5C-D). Thus, the pharmacological inhibition of PPARγ and *CEPT1* knockdown further compromised Olig2-OPC differentiation and maturation. To further explore the potential interaction of Olig2 and PPARγ, analysis of the protein-protein interactions (PPIs) in RNA-seq data revealed that SMARCA4/Brg1 might act as an intermediate factor involved in the interaction between Olig2 and PPARγ (Figure 5E). qPCR results further confirmed the upregulation of SMARCA4/Brg1 expression in Olig2-OPCs (Figure 5F). Thus, we proposed that overexpression of Olig2 might promote OL differentiation partially through the SMARCA4/Brg1-PPARG-CEPT1 signaling axis.

### Transplanted Olig2-OPCs promoted neuronal survival by suppressing inflammation and the immune response in ischemic stroke rats

**Figure 6:**
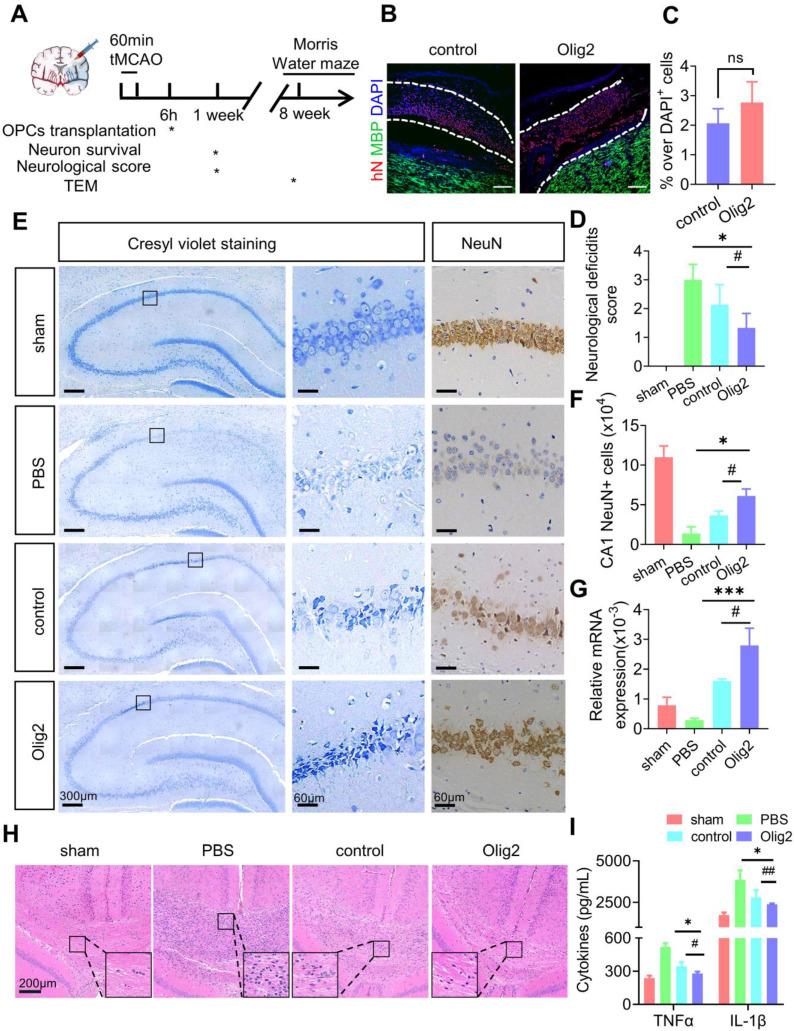
** Transplanted Olig2-OPCs promoted neuronal survival by suppressing inflammation and the immune response in ischemic stroke rats. A** Scheme depicting the procedure of OPC or PBS administration after tMCAO and the detection of neuronal survival, neurological deficit scores, TEM and Morris water maze test. **B** Representative images showing that at 1 week after transplantation, transplanted cells were identified by human nuclei (hN) staining. Notably, the majority of the transplanted cells were found around the hippocampus. Scale bar, 200 μm. **C** Quantitative results from brain sections containing structures of the hippocampus showed that the engraftment efficiency was indistinguishable between the Olig2-OPC and control-OPC transplantation groups (ns p>0.01, by a two-tailed Student's t test). **D** Neurological deficit scores in different groups at 1 week were evaluated (n = 11, * p < 0.05, comparison between the sham group versus the groups that received cell transplantation; # p < 0.05, by one-way ANOVA, comparison between the two groups that received cell transplantation, by one-way ANOVA). **E** Representatives of cresyl violet staining and NeuN DAB staining performed on the sections from the hippocampus in the sham, PBS and groups that received cell transplants subjected to 8 weeks of reperfusion after 60 min of tMCAO with PBS control and intraventricular administration of Olig2-OPCs or control-OPCs. The squared areas in cresyl violet staining are shown at high magnification (scale bar, 60 μm). Corresponding areas are also shown for NeuN staining (scale bar, 60 μm). **F** The number of hippocampal CA1 neurons in the different groups measured by stereological analysis (n = 3 for each group, * p < 0.05, comparison between the sham group and the groups that received cell transplantation; # p < 0.05, one-way ANOVA, comparison between the two groups that received cell transplantation, one-way ANOVA). **G** qPCR analysis of *BDNF* mRNA expression levels from the sham, PBS and groups that received cell transplantation (n = 3, *** p < 0.001, comparison between the sham and the groups that received cell transplantation; # p < 0.05, by one-way ANOVA, comparison between the two groups that received cell transplantation, by one-way ANOVA). **H** Representative tissue sections of H&E staining in the hippocampus at 1 week from the sham, PBS and groups that received cell transplantation. Scale bar, 200 μm. **I** ELISA of TNF-α and IL-1β in the infarct brain tissue from the sham group and groups that received cell transplantation or PBS group (n = 3, * p < 0.05, comparison between the sham group versus the group that received cell transplantation; # p < 0.05, ## p < 0.01, by one-way ANOVA, comparison between the two groups that received cell transplantation, by one-way ANOVA). The graphs represent the individual data points and the mean ± SEM of three independent experiments.

To investigate the potential therapeutic benefit of Olig2-OPCs, we used a transient middle cerebral artery occlusion (tMCAO) rat model of ischemic stroke, Olig2-OPCs, and control-OPCs were frozen, thawed, and allowed to recover for 24-48 h before transplantation. Then, Olig2-OPCs, control-OPCs and PBS were intracerebroventricularly injected 6 h after tMCAO (Figure 6A). Some animals were sacrificed at 1 week, at which point human nuclear (hN) cells were distributed throughout the hippocampus and grafted well (Figure 6B). As shown in Figure 6C, the engraftment efficiency was indistinguishable between the Olig2-OPC and control-OPC transplantation groups, and we did not observe the presence of cell clusters or overt tumorigenesis in these two groups. In addition, at the same time point, no hN or MBP -colabeled cells were observed, indicating that the implanted OPCs did not differentiate into myelinating cells. On that basis, neurological deficits were first evaluated using the Menzies score as previously described 29, and a greater improvement in neurological deficits was detected in both the Olig2-OPC and control-OPC groups than in the PBS groups at 1 week posttransplantation.

Notably, the rats in the Olig2-OPC group showed more improvement than the rats in the control-OPC group (Figure 6D). We then evaluated neuronal survival of the CA1 region of the brain following tMCAO with and without cell infusion, and cresyl violet staining and immunohistochemistry for NeuN were performed. Dramatic neuronal loss in the CA1 region by 1 week after transplantation was observed in the PBS group of rats. The number of NeuN^+^ neurons was assessed, and both types of OPCs showed significantly better protection of neurons than PBS. Most importantly, Olig2-OPCs exhibited a better neuroprotective effect than control-OPCs (Figure 6E-F). We also found that transplanted OPCs strongly increased *BDNF* mRNA expression in rats that received Olig2-OPC transplantation (Figure 6G), which might contribute to the protective effects on neurons against ischemic insult 30, 31.

OPCs have recently been shown to regulate inflammatory responses. Histological analysis of the hippocampus of rats revealed a large aggregation of mononuclear immune cells in tMCAO rats; however, these cells were significantly reduced after cell infusion, especially in the group that received Olig2-OPC transplantation (Figure 6H). We next examined the proinflammatory cytokines tumor necrosis factor-α (TNF-α) and other types of interleukins, such as IL-1β, which were secreted in the infarcted area of the brain tissue of tMCAO rats. The tMCAO model triggered significantly increased secretion of TNF-α and IL-1β. However, the levels of these proinflammatory cytokines showed marked drops after cell transplantation. Notably, Olig2-OPCs exhibited a better anti-inflammatory effect than control-OPCs (Figure 6I). These findings suggested that OPC infusion could rapidly improve neurological function at the early stage and protect neurons from death by suppressing inflammation and the immune response, while infusion of Olig2-OPCs displayed superior therapeutic potential in rats with ischemic stroke.

### Enhanced OL generation and remyelination by Olig2-OPC transplantation promoted the recovery of spatial learning and cognitive ability

**Figure 7:**
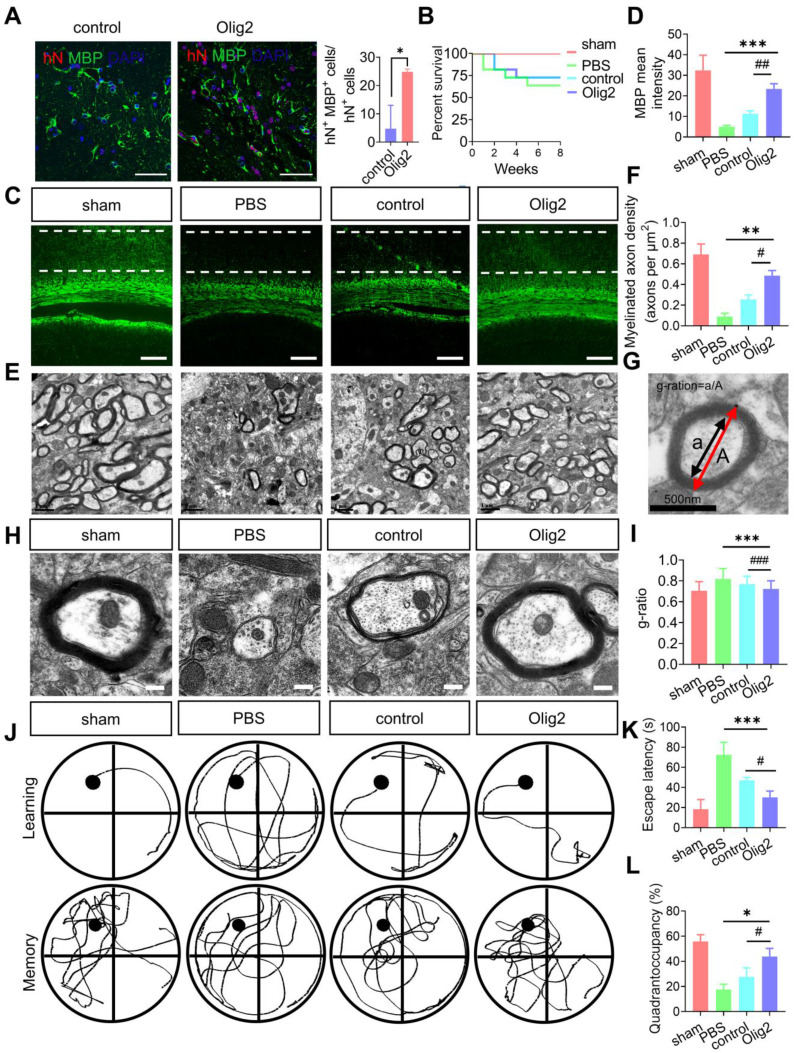
** Enhanced OL generation and remyelination by Olig2-OPC transplantation promoted the recovery of spatial learning and cognitive ability. A** Representative immunofluorescent staining of hippocampal sections in the sham, PBS and groups that received cell transplantation (scale bar, 100 μm). Donor-derived myelination defined by MBP staining (green) was apparent throughout the hippocampus in rats receiving Olig2-OPCs at 8 weeks. In contrast, very few MBP^+^ cells expressed hN in animals receiving control-OPC transplantation (scale bar, 100 μm, * p < 0.05, by a two-tailed Student's t test). **B** Analysis of survival (percentage, Kaplan-Meier) from the sham and PBS groups and groups that received cell transplantation. **C** Representative immunofluorescent images of MBP in the sham, PBS and groups that received cell transplantation (scale bar, 100 μm). **D** Quantitative analysis of the fluorescence intensity of MBP staining in the CA1 region (*** p < 0.001, comparison between the sham group and the group that received cell transplantation; ## p < 0.01, by one-way ANOVA, comparison between the two groups that received cell transplants, by one-way ANOVA). **E** Representative electron micrographs of brain sections from the sham, PBS and groups that received cell transplantation (scale bar, 1 μm). **F** Quantitative analysis of the density of myelinated axons from sham, PBS and groups that received control-OPC and Olig2-OPC transplantation (** p < 0.01, comparison between the sham versus groups that received cell transplants; # p < 0.05, by one-way ANOVA, comparison between the two groups that received cell transplantation, by one-way ANOVA). **G** Diagram and analysis of the average g-ratios of myelinated axons. Line “A” indicates the diameter of a myelinated axon fiber, and line “a” indicates the diameter of the axonal caliber. Scale bar, 0.5 μm. **H** Representative electron micrographs at high magnification. Scale bars, 0.2 μm.** I** Mean g ratio of the four groups (n = 100, *** p < 0.001, comparison between the sham group and the group that received cell transplantation; ### p < 0.001, by one-way ANOVA, comparison between the two groups that received cell transplantation, by two-way ANOVA). **J** Representative sample paths from the maze trials (upper panel) and the search patterns on the probe trials (lower panel) after 8 weeks. **K** The Morris water maze test was performed to determine the spatial learning ability of the four groups, as shown by the time (escape latency) to find the submerged platform after 8 weeks (n = 6/7, *** p < 0.001, comparison between the sham group and the group that received cell transplantation; # p < 0.05, by two-way ANOVA, comparison between the two groups that received cell transplantation, by one-way ANOVA). **L** Probe trials were performed 4 h after the last maze trials and monitored by relative radial quadrant occupancy (time spent in the target quadrant, n = 6/7, * p < 0.05, comparison between the sham group versus the group that received cell transplantation; # p < 0.05, by one-way ANOVA, comparison between the two groups that received cell transplantation, by one-way ANOVA). The graphs represent the individual data points and the mean ± SEM of three independent experiments.

Given the effect of Olig2 overexpression on OPC/OL differentiation and maturation *in vitro*, we then investigated whether transplanted Olig2-OPCs promoted long-term remyelination *in vivo*. Transplanted cells were double labeled with hN and partly differentiated into MBP^+^ OLs after 8 weeks. Notably, more hN^+^ MBP^+^ OLs were observed in the rats receiving Olig2-OPC transplants than in control-OPCs, suggesting that Olig2-OPCs had better engraftment than the control-OPC group (Figure 7A). Remarkably, the OPC-transplanted mice displayed greatly prolonged survival, with reduced death over 8-week period of observation, while there was no significant difference between control-OPC and Olig2-OPC transplantation (Figure 7B). Quantification of the fluorescence intensity of MBP staining revealed that both OPCs significantly preserved myelin integrity, and Olig2-OPCs showed protective effects superior to those of control-OPCs (Figure 7C-D). In light of the markedly enhanced myelin integrity, we next sought to confirm that this effect was associated with the formation of ultrastructurally compact myelin around host axons by transplanted OPCs. Transmission electron microscopy (TEM) was used to evaluate the ultrastructure of myelinated axons. We found that most of the axons lost the myelin sheath in the PBS group, and the control-OPC group showed slight remyelination. However, the rats in the Olig2-OPC group had more myelinated axons (Figure 7E-F). The G-ratio, which was the ratio of the axon diameter to the total diameter of a myelinated fiber (Figure 7G), was evaluated to assess the density of myelinated axons. Compared to the control-OPC group, the Olig2-OPC group had significantly lower g-ratio values, indicating that a thicker myelin sheath was generated (Figure 7H-I). The effects of the grafted Olig2-OPCs on the density of myelinated axons and the lower g ratio value might indicate their beneficial impact on axons.

To further examine whether Olig2-OPC transplantation could improve spatial learning and memory in rats with ischemic stroke, we utilized the Morris water maze to examine learning and memory at 8 weeks after tMCAO. As shown in Figure 7J-K, the rats in the PBS group required more time to find the platform than those in the sham-treated group; however, rats in both the Olig2-OPC group and the control-OPC group had a lower escape latency than rats in the PBS group. Interestingly, rats in the Olig2-OPC group showed better performance than those in the control-OPC group. The rats in the sham-treated group and both OPC-transplanted groups spent significantly more time in the target quadrant than those in the PBS group. Notably, the animals in the Olig2-OPC group spent more time in the quadrant than those in the control-OPC group (Figure 7L). These results indicated that the transplanted Olig2-OPCs were superior to the control-OPCs in the recovery of learning and memory. In summary, Olig2-OPC transplantation efficiently promoted myelination, resulting in recovery of learning and memory defects in rats with ischemic stroke.

## Discussion

Although previous researchers have shown that neuronal cell death within the gray matter is the main pathological characteristic of ischemic stroke, a recent study highlighted the equal importance of white matter integrity in long-term recovery after ischemic stroke 32. Thus, the regulation of remyelination within white matter may provide a novel therapeutic target for the treatment of ischemic stroke 33. In particular, hiPSC-derived OPC transplantation has shown significant therapeutic potential in many biomedical studies for myelin repair 34-36. We reported the generation of Olig2-OPCs derived from hiPSCs through sequential application of GANT61 and overexpression of Olig2. Olig2-OPC transplantation reduced neuronal death, promoted remyelination and eventually improved long-term cognitive and memory deficits in a rat model of ischemic stroke.

A key prerequisite for biomedical applications of human OPC replacement therapy is establishing expandable and effective protocols to generate human OPCs from hiPSCs. The differentiation of hiPSCs into OPCs is controlled by the interaction of major TFs with cis-acting elements to direct the downstream transcription of partner genes 37. To the best of our knowledge, several TFs (*SOX9, SOX10*,* ZFP488*) and TF combinations (*NKX6.2, OLIG2,* and *SOX10*) have been reported to facilitate rapid late OPC differentiation from hiPSCs *in vitro* 12, 16, 17, 38, 39. In the present study, GANT61, a small molecule inhibitor, was applied to transiently inhibit Shh signaling to obtain NPCs from hiPSCs before the later gliogenesis stage. The generated GANT61-NPCs were sequentially differentiated into a considerable number of OPCs by overexpression of Olig2. As shown in other studies, the Shh signaling pathway is required to induce the proneural gene *NGN2* and establish combinatorial dorsal-ventral TF codes for MN specification 40, 41. Samanta et al. 20 reported that GANT61 treatment drove a subset of the Shh-responsive pool of endogenous NPCs within the subventricular zone (SVZ) to differentiate into oligodendrocytes by inhibiting GLI1 expression *in vivo*. Consistently, our laboratory has also reported that temporal and partial inhibition of Shh signaling by GANT61 in hiPSC-derived NSCs results in early OL maturation *in vitro*. Thus, inhibition of Shh signaling may facilitate OL differentiation. Given that no further studies have identified detailed mechanisms to explain why temporal Shh inhibition leads to OL generation, our present data showed that Olig2 was dephosphorylated at Ser147 in GANT61-NPCs, indicating that GANT61 interfered with the posttranslational modification of Olig2. Huiliang Li et al. reported that Ser147 in the domain of Olig2 was phosphorylated during MN production and dephosphorylated at the onset of oligodendrogenesis 27. We speculated that GANT61 treatment resulted in suppression of MN development by blocking the phosphorylation of Olig2 Ser147. Furthermore, dephosphorylated Olig2 preferentially formed form heterodimers with the MN-specific TF *NGN2*. Indeed, the mRNA expression level of *NGN2* was significantly decreased in GANT61-NPCs. MNs in the spinal cord did not form normally in *NGN2*-deficient mice 42. We believe that GANT61-induced downregulation of *NGN2* expression reduces the amount of *NGN2* available for activating MN-specific genes and that Olig2 cannot later act in concert with *NGN2*, which is essential for MN specification 43, 44. Another study showed that the mRNA expression level of *HB9*, a downstream TF of *NGN2* 27, was also decreased in GANT61-NPCs. Thus, Shh inhibition resulted in suppression of MN development, which led to dysfunction of Olig2 in regulating MN development, went straight to OLs, and finally facilitated OPC differentiation in cooperation with Olig2 overexpression.

Next, we demonstrated that Olig2 overexpression could substantially accelerate oligodendroglial lineage commitment 45, 46. *GPR17* was transiently expressed in early NG2^+^ OPCs and completely disappeared before cells reached terminal maturation 47. Our RNA-seq data indicated that Olig2-OPCs expressed lower levels of *GPR17* than control-OPCs (Figure S6B, TPM, 0.27 vs. 0.87, p < 0.05), further showing that Olig2 overexpression progressively downregulated *GPR17* expression to allow early NG2^+^ cells to complete maturation. This phenomenon was similar to that of our subsequent data, as revealed by flow cytometry analysis for O4 expression, which is a specific mature OL marker 48. Olig2-OPCs showed a comparable ability to differentiate and mature into O4^+^ OLs compared to the control group. In addition, we also observed that the transcriptional level of* OLIG1* did not change with Olig2 overexpression (Figure S9). This finding suggested that Olig2-induced OPC differentiation was independent of *OLIG1* transcription. Indeed, consistent with a previous study, *OLIG1* and *OLIG2* have nonoverlapping developmental functions in patterning and the formation of OLs. It appeared that *OLIG1* and *OLIG2* have distinct biological functions that could be separable from those of each other during OL development. However, the mRNA expression level of *NKX2.2* was significantly upregulated after Olig2 was overexpressed. It was believed that Olig2 overexpression induced the expression of *NKX2.2*. Therefore,* NKX2.2* upregulation might function downstream of the *OLIG2* regulatory network during OL lineage specification. Sun *et al.* also reported that Olig2 and *NKX2.2* physically interacted with each other 49. However, it was not clear how *OLIG2* and* NKX2.2* normally work together in OL specification.

The contribution of phospholipids and their derivatives to the CNS is crucial, and many articles have demonstrated their roles in both health and disease 50; however, the phospholipid synthesis pathways involved in OL generation and myelination have rarely been reported. Here, significant metabolic remodeling of phospholipid synthesis was observed in Olig2-OPCs, suggesting that enhanced phospholipid synthesis might contribute to the Olig2-OPC functional phenotype. We also showed that Olig2 overexpression altered the expression of genes involved in the PPARγ signaling pathway, which are important in whole-body energy metabolism and collectively involved in lipid metabolism 51. Consistent with findings from previous studies, PPARγ controlled lipid metabolism to induce OL differentiation, and a specific PPARγ antagonist suppressed oligodendrocytic maturation 52. Hence, PPARγ might act as a putative positive regulator of Olig2-OPC differentiation and maturation by affecting phospholipid metabolic reprogramming, but Olig2 did not interact with PPARγ. In fact, we found that the chromatin remodeling factor SMARCA4/Brg1 might act as a bridge between Olig2 and PPARγ, which was responsible for CEPT1-mediated phospholipogenesis by PPI analysis. Other laboratories have also reported that Olig2 targets Brg1 to initiate OL differentiation 53, 54, and Brg1 expression was found to be upstream of PPARγ and Shh expression in basal cells from the ureteral epithelium 55. In another study, Brg1 was shown to act on the PPARγ2 locus to promote adipogenic differentiation 56. Nevertheless, we believe that overexpression of Olig2 promotes OL differentiation partially through the SMARCA4/Brg1-PPARG-CEPT1 signaling axis, but the detailed molecular mechanisms remain to be further investigated.

We then focused on the functional assessment of Olig2-OPCs and demonstrated their *in vivo* therapeutic potential in rats with ischemic stroke. We observed that Olig2-OPCs could better protect host neurons from death in an ischemic environment by suppressing inflammation and the immune response. In the present study, although we did not present direct evidence that PPARγ signaling or PPARγ-mediated phospholipogenesis was responsible for the therapeutic efficacy of OPC transplantation, we found that infused Olig2-OPCs could reduce the release of TNFα and IL-1β following ischemic injury. These results indicated that Olig2-OPCs inhibited inflammation partly through PPARγ signaling. Indeed, several *in vitro* studies have provided detailed insights into the mechanism that underlies the neuroprotective actions of upregulation of PPARγ signaling following ischemic injury 57. Furthermore, PPARγ and its agonists not only reduce immune reactions outside the nervous system but also reveal powerful anti-inflammatory potential in ischemic brains. Consistent with neuronal PPARγ contributing to neuroprotection, thiadiazolidinone PPARγ agonists also prevent the apoptosis of primary cortical neurons evoked by cell-free supernatant from lipopolysaccharide (LPS)-activated microglia 58. Most importantly, although there are multiple targets for PPARγ signaling in ischemic injury, we first report that activated phospholipid biosynthesis might play an important role in the inhibition of inflammation. This phenomenon was consistent with previous findings, in which phospholipids and lipid derivatives were considered potential neuroprotective compounds 59. Therefore, to a certain extent, the anti-inflammatory effect of OPC-based cell therapy for stroke might be attributed to PPARγ-mediated phospholipid metabolic reprogramming.

We also provided comparable evidence that transplanted Olig2-OPCs significantly enhanced MBP^+^ OL differentiation and maturation, thereby promoting remyelination in the hippocampus. Myelination is inhibited in the brain after stroke, and migrating OPCs from outside the ischemic region have difficulty myelinating spontaneously 60. Substantial evidence has shown that myelin degeneration and diminished renewal could cause cognitive and spatial memory loss, but promotion of oligodendroglial differentiation and myelination rescued this decline 61-63. Indeed, transplanted Olig2-OPCs significantly protected cognitive and spatial memory, as shown by the Morris water maze test in the animal experiments. We strongly believe that Olig2-OPC transplantation rescued learning and memory loss partially by enhancing the remyelination process in rats with ischemic stroke.

In conclusion, we successfully generated human OPCs by sequentially applying GANT61 and overexpressing Olig2 from hiPSCs. We showed that Olig2-OPCs increased the gene expression of the PPARγ signaling pathway, and activated CEPT1-mediated phospholipogenesis, Strikingly, we also found that transplantation of Olig2-OPCs could enhance the recovery of learning and memory deficits by promoting neuron survival and increasing remyelination in rats with ischemic stroke. Thus, Olig2-OPC transplantation might represent an ideal cell resource for cell-based therapy for ischemic stroke.

## Materials and methods

### Generation of hiPSC cell lines with induced expression of Olig2

Human Olig2 cDNA (Hanbio, China) was inserted into the Tet-inducible plasmid pLox and cotransfected with pSALK-cre into hiPSCs (provided by Dr. Jiekai Chen, Guangzhou Institutes of Biomedicine and Health Chinese Academy of Science, China) 64 by electroporation (800 v, 3 μF) with Gene Pulser Xcell (Bio-Rad, Hercules, CA, USA). Stably transfected cells were isolated by selection with neomycin (G418, 250 μg/mL, Thermo Fisher Scientific, MA, USA) and were confirmed for successful doxycycline (Selleck, Houston, TX, USA)-induced Olig2 overexpression by western blotting and named Olig2/hiPSCs.

### Olig2/hiPSC culture and neural induction

Olig2/hiPSCs were maintained in feeder free conditions using mTeSR1 medium (Stem Cell Technologies, Canada) on hESC-qualified Matrigel (Corning, USA), which was split twice a week using ReLesR (Stem Cell Technologies, Canada). NPCs were differentiated from Olig2/hiPSCs by modifying previously described protocols 39, 65. Specifically, Olig2/hiPSCs at passages 20-30 were enzymatically and mechanically sectioned and detached. The diluted colonies were replated into a six-well plate, and neural induction medium 1 (NIM1) supplemented with GANT61 (5 μM; Abmore, USA) was added for 2 days. The culture medium was then switched to neural induction medium 2 (NIM2) supplemented with GANT61 for another 5 days. The generated NPCs (GANT61-NPCs) were then split into Matrigel-coated six-well plates at ratios of 1:15-1:20 by treatment with Accutase (Sigma-Aldrich, USA) and cultured NPC maintenance medium (NSMM) supplemented with Y-27632 (10 μΜ, Med Chem Express, USA). For the NIM1, NIM2, and NSMM detailed compositions used, see Supplementary Table S1.

### Oligodendroglia differentiation

For OL lineage differentiation, a two-step differentiation protocol was utilized. Specifically, expanded GANT61-NPCs were plated at 500,000 cells/well on poly-L-ornithine/laminin (Sigma-Aldrich, USA)-coated six-well plates. The day after passage, the culture medium was changed to GIM supplemented with doxycycline (1 μg/mL, Med Chem Express, USA). The GIM was changed every other day. After 4 days, the GIM was replaced with DM supplemented with the same components but lacking PDGF-AA and SAG, and the medium was changed every other day. For the GIM and DM detailed compositions used, see Supplementary Table S1.

### Immunofluorescence staining and immunohistochemical staining

Cells were fixed with 4% paraformaldehyde for 15 min at room temperature, and brain sections were processed for immunofluorescence staining. After fixation, the coverslips were washed with PBS three times, blocked with 4% bovine serum albumin (BSA) for 30 min at 37 °C and then incubated with the corresponding primary antibody overnight. The next day, the coverslips were incubated with fluorescence-conjugated secondary antibodies (Invitrogen, San Diego, CA, USA) for 1 h at room temperature in the dark. Coverslips were washed three times with PBS, stained with 4′,6-diamidino-2-phenylindole (Vector Laboratories, Burlingame, CA) for at least 10 min, washed with PBS three times, and mounted with mounting medium (Invitrogen, San Diego, CA, USA). Cells on the coverslip were imaged using a fluorescence microscope (Eclipse Ti2-U, Nikon, Japan). All related primary antibodies in this article are given in Supplementary Table S2.

### Protein and mRNA analysis

For immunoblotting, whole cell lysates were extracted with lysis buffer (Beyotime, China) supplemented with protease inhibitors (Roche, UK) and then concentrated with a BCA protein assay kit (Thermo Fisher Scientific, USA). Fifty micrograms of the total protein were loaded into each well and separated by 10% SDS-PAGE electrophoresis. The expression of all proteins in this article was probed with a primary antibody overnight. Proteins were normalized to the housekeeping protein GAPDH or β-actin protein in the same blotted sample.

For qPCR, TRIzol (Invitrogen, USA) was used to extract total RNA. cDNA was synthesized using High Capability RNA-to-cDNA Mater Mix (Transgen Biotech, China). qPCR was performed using SYBR Green PCR Master Mix (Transgen Biotech, China). Primer sequences are provided in Supplementary Table S3.

For ELISA assay, brain homogenate from the infarct sites was obtained using a tissue homogenizer and centrifuged at 12000 rpm for 20 min; the supernatant was retained. Then, the concentrations of inflammatory factors, including TNFα and IL-1β, were determined using ELISA kits (Dakewe, Beijing, China) according to the specifications.

### Flow cytometry analysis

Cells were enzymatically harvested using Accutase, centrifuged and resuspended in 100 μL of FACS buffer (1X PBS, 2% fetal bovine serum and 0.02% sodium azide). To determine NG2, PDGFRa, and O4 expression, Olig2-induced progenies were incubated with 1/20 dilutions of NG2-PE, PDGFRa-APC, and O4-APC antibodies or unstained control for 15 min at 4 °C, washed and resuspended in 150 μL of FACS buffer. Cells were analyzed on a BD flow cytometer. After exclusion of cell doublets, gates were established based on the corresponding unstained controls (Figure S8B-C). All flow cytometric data were further analyzed using BD Cyto Expert software (BD Biosciences, San Jose, CA, USA).

### NG2^+^ cell purification and cryopreservation

The induction cultures were maintained for 7 days in OL differentiation medium and purified using fluorescence-activated cell sorting (FACS) with an anti-NG2-PE antibody, following the manufacturer's instructions. Purity was checked in all cases by flow cytometry; > 93% NG2^+^ purity of the isolated population was obtained. Purified NG2^+^ cells were used for transcriptome and metabolome analysis, or cryopreserved until further cell transplantation. For cryopreservation, the NG2^+^ purified fraction was resuspended in OL DM and mixed 1:1 with ice-cold Pro-Freeze medium (Lonza, USA) containing 15% DMSO. Cells were immediately stored in a freezing container at -80 °C overnight and transferred the next day to liquid nitrogen for long-term storage. Sorting efficiency was determined by flow cytometry. Specifically, the cell aliquot was resuspended in 95 μL of ice-cold PBS, added to 5 μL of NG2-PE antibody and incubated for 15 min in the refrigerator. Then, the cells were washed with 2 mL of PBS, centrifuged, resuspended in 200 μL of PBS and analyzed on a flow cytometer (BD Biosciences, USA). Proper unstained control samples were used to define gate thresholds (Figure S8A).

### Lentiviral-mediated short-hairpin RNA (shRNA) interference of *CEPT1*

shRNAs (sh*RNA1*, sh*RNA2* and sh*RNA3*, see Supplementary Table S4) designed to knock down *CEPT1* synthesis were annealed and cloned into the EcoRI-XhoI sites of the lentiviral vector SF-LV-shRNA-eGFP. The shRNA lentivirus was produced by calcium phosphate-mediated transient transfection of HEK-293T cells by cotransfecting 20 μg of lentiviral plasmid, 15 μg of packaging plasmid (psPAX2) and 6 μg of coat protein plasmid (pMD2.G). Virus was collected from the culture supernatants on d2 and d3 posttransfection. Olig2-OPCs were transduced with concentrated lentiviruses at an MOI of 20. The efficacy of *CEPT1* knockdown was assessed after 2 days by qPCR or western blot.

### Neurological deficits score

The evaluation of neurological deficits was performed at 1 week posttransplantation: 0 = no deficit; 1 = forelimb weakness; 2 = circling to the affected side; 3 = partial paralysis on affected side; and 4 = no spontaneous motor activity as described previously 29, 66.

### TEM

The rats were sacrificed and perfused overnight in Karnovsky's fixative. The brain samples were fixed in 2.5% glutaraldehyde for 2 h and sectioned as described previously 67. TEM images were captured using a high-resolution charge-coupled device (CCD) camera. Images were processed using a digital micrograph (Gatan, USA). G-ratios were analyzed using Image J software (NIH, USA), and ~100 remyelinated axons were measured for each group.

### A rat model of tMCAO and transplantation

All animal experimental procedures and transplantations were conducted following protocols approved by the Sun Yat-sen University Animal Use and Care Committee. To induce tMCAO 68, a total of 50 Sprague-Dawley male rats (180-200 g), including 6 animals excluded from further experiments (due to unsuccessful surgery), were anesthetized with 1.5% isoflurane in a 30% O_2_/69% N_2_O mixture and a silicone-coated filament was inserted into the external carotid artery (ECA) and passed through the internal carotid artery (ICA) to block the middle carotid artery (MCA) 69. The sham-operated rats underwent the same isoflurane anesthesia and surgical procedures as the tMCAO group except the insertion of an intraluminal filament. To inject a large volume of cell suspension and avoid swelling introduced by the grafted cell suspension, we performed intracerebroventricular injection. Both Olig2-OPCs and control-OPCs were suspended at a final concentration of 100,000 cells per μL in PBS, and then 5 μL of cells was administered to the left cerebral ventricles of rats 6 h after tMCAO. Control rats were infused with PBS as a vehicle control. One week or eight weeks after transplantation or PBS injection, the brains were processed for immunohistochemistry or TEM. For histological assessment, the dissected brain sections were stained with 0.01% (w/v) cresyl violet for 10 min, followed by graded ethanol dehydration, and additional sections were used for hematoxylin and eosin (H&E) staining.

### Morris water maze test

Eight weeks after tMCAO, the Morris water-maze test was performed 70, 71. Rats were placed in the water by hand, with each animal facing the wall at one random start location out of four, and they were allowed to find the submerged platform within 90 s. A trial was terminated when the rats found the platform. If the rat did not find the hidden platform within 90 s, it was guided onto the platform with a stick. The rat was allowed to stay on the platform for 15 s before being removed. The training was repeated from each of the four randomized starting locations, and 4 h was allowed between sessions. The latency time and swimming distance were monitored by an overhead video camera and analyzed by an automated tracking system (Noldus, Netherlands). One hour after the final training trial, each rat was subjected to a probe trial (90 s) in which no platform was present. The rat was placed in the water at the same random start location, and the time spent in the quadrant that formerly contained the platform was recorded to assess the level of spatial bias.

### RNA-seq analysis

Total RNA was extracted from sorted control-OPCs and Olig2-OPCs following a standard protocol with TRIzol reagent (Life Technologies, CA, USA) followed by RNA library preparation with the Illumina Tre Seq strand-specific mRNA sample preparation system. All RNA-seq libraries were sequenced with a read length of single-end 75 bp using the Illumina Next Seq 500 and a final read length of over 45 million reads per sample.

### Metabolome profiling

One milliliter of 80% methanol was added to sorted control-OPCs and Olig2-OPCs, incubated for 30 min at 1500 rpm at 4 °C, and then centrifuged for 10 min at 12000 rpm at 4 °C. The supernatant was transferred to a clean 1.5-mL centrifuge tube, and dried using Speed Vac (Tegent Scientific, England). The dried extracts were redissolved in 1% acetonitrile in water, and the upper layer of liquids was collected for LC-MS analysis. The LC-MS parameters referenced a previous method 72. A column ACQUITY UPLC HSS T3 1.8 μm, 2.1 × 100 mm column (Waters, Ireland) was adopted in the present study. Ultra-performance liquid chromatography (Agilent 1290 II, Agilent Technologies, Germany) coupled to 5600 Triple Quadrupole-TOF MS (AB Sciex, Singapore) was applied to acquire metabolome data. The MS parameters for detection were as follows: ESI source voltage -4.5 kV; vaporizer temperature, 500 °C; drying gas (N_2_) pressure, 50 psi; nebulizer gas (N_2_) pressure, 50 psi; curtain gas (N_2_) pressure, 35 psi; and scan range m/z 60-600. Information-dependent acquisition mode was used for MS/MS analyses of the metabolites. The collision energy was set at 35 ± 15 eV. Data acquisition and processing were performed using Analyst® TF 1.7.1 Software (AB Sciex, Canada). All detected ions were extracted using Marker View 1.3 (AB Sciex, Canada) in Excel in the format of a two-dimensional matrix, including the mass to charge ratio (m/z), retention time, and peak areas, and isotopic peaks were filtered. Peak View 2.2 (AB Sciex, Canada) was applied to extract MS/MS data and perform comparisons with metabolite databases (AB Sciex, Canada), HMDB, METLIN, and standard references to annotate ion IDs. The self-compiled R program was used for statistical analysis.

### Data analysis and statistics

PRISM™ 8.0.2 for Windows (GraphPad) was used for the statistical analysis. All data represent the mean ± SEM. All other assessments were analyzed using Student's *t* test when only two groups were compared or one-way ANOVA when three or more groups were compared.

## Supplementary Material

Supplementary figures and tables.

Supplementary table of differential metabolites.

## Abbreviations

CNS: central nervous system
ECA: external carotid artery
ESCs: human embryonic stem cells
H&E: hematoxylin and eosin
FACS: fluorescence-activated cell sorting
hiPSCs: human induced pluripotent stem cells
ICA: internal carotid artery
MNs: motor neurons
MAG: myelin-associated glycoprotein
MOG: myelin oligodendrocyte glycoprotein
MCA: middle carotid artery
NG2: neural/glial antigen 2
NPCs: neural progenitor cells
OL: oligodendrocyte
OPCs: OL progenitor cells
PDGFRα: platelet-derived growth factor α
PC: phosphatidylcholine
PE: phosphatidylethanolamine
PLP1: proteolipid 1
PPARγ: peroxisome proliferator-activated receptor γ
Shh: sonic hedgehog
TEM: transmission electron microscopy
tMCAO: transient middle cerebral artery occlusion
