# Supplementary material for: Shh and Olig2 sequentially regulate oligodendrocyte differentiation from hiPSCs for the treatment of ischemic stroke
Source: Theranostics. 2022 Mar 28;12(7):3131–49. doi: 10.7150/thno.69217 (PMC9065175; doi:10.7150/thno.69217)
Supplement: Supplementary file 1 — Supplementary figures and tables. [file thnov12p3131s1.pdf]

Supplementary materials for

Shh and Olig2 sequentially regulate oligodendrocyte differentiation from hiPSCs for the treatment of ischemic stroke

Jian Xu, Jingxin Zhao, Rui Wang, Yidi Zhang, Lan Shen, Qian Xiao, Yuan Xie, Jinjun Jiang, Yichu Nie, Wenbin Deng

The file includes:

- Supplementary Table S1. Detailed composition of culture medium.
- Supplementary Table S2. Primary antibodies used for immunostainings.
- Supplementary Table S3. Sequence of primers for qPCR analysis.
- Supplementary Table S4. Sequences of shRNA assays.
- Supplementary figures and figure legends

Supplemental Tables

Table S1. Detailed composition of culture medium

Media	Components	Provider	Final con.
**NIM1**	DMEM/F12	Life Technologies	50%
	Neurobasal	Gibco	50%
	B27 Supplement (50X)	Stem cell technology	1X
	N2 Supplement (100X)	Stem cell technology	1X
	Recombinant hLIF	PEPROTech	10 ng/ml
	SB431542	Med Chem Express	2 μM
	CHIR99021	Med Chem Express	3 μM
	GlutaMAX	Life Technologies	2 mM
	Compound E	Med Chem Express	0.1 μM
	Dorsomorphin	Med Chem Express	2μM
**NIM2**	DMEM/F12	Life Technologies	50%
	Neurobasal	Gibco	50%
	B27 Supplement (50X)	Stem cell technology	1X
	N2 Supplement (100X)	Stem cell technology	1X
	Recombinant hLIF	PEPROTech	10 ng/ml
	SB431542	Med Chem Express	2 μM
	CHIR99021	Med Chem Express	3 μM
	GlutaMAX	Life Technologies	2 mM
	Compound E	Med Chem Express	0.1 μM
**NSMM**	DMEM/F12	Life Technologies	50%
	Neurobasal	Gibco	50%
	B27 Supplement (50X)	Stem cell technology	1X
	N2 Supplement (100X)	Stem cell technology	1X
	Recombinant hLIF	PEPROTech	10 ng/ml
	SB431542	Med Chem Express	2 μM
	CHIR99021	Med Chem Express	3 μM
	GlutaMAX	Life Technologies	2 mM

**GIM**	DMEM/F12	Gibco	
	B27 supplement lacking vitamin A (50X)	Stem cell technologies	1X
	N2 Supplement (100X)	Stem cell technologies	1X
	penicillin/streptomycin	Gibco	1%
	SAG	Med Chem Express	1 µM
	PDGF-AA	R&D Systems	10 ng/ml
	NT-3	Millipore	10 ng/ml
	IGF-I	R&D Systems	10 ng/ml
	AA	Sigma–Aldrich	200 µM
	T3	Sigma–Aldrich	60 ng/ml
**DM**	DMEM/F12	Gibco	
	B27 supplement lacking vitamin A (50X)	Stem cell technologies	1X
	N2 Supplement (100X)	Stem cell technologies	1X
	penicillin/streptomycin	Gibco	1%
	NT-3	Millipore	10 ng/ml
	IGF-I	R&D Systems	10 ng/ml
	AA	Sigma–Aldrich	200 µM
	T3	Sigma–Aldrich	60 ng/ml
	dbcAMP	Sigma–Aldrich	100 µM

Table S2. Primary antibodies used for immunostainings

Antigen	Dilution	Reference	
Olig2	1/100	Rabbit IgG	ab254043
NESTIN	1/3200	Mouse IgG	CST#33475
SOX2	1/400	Mouse IgG	CST#3579
O4	1/500	Mouse IgM	R&D MAB1326
O4-APC	1/50	Mouse IgG	Miltenyi Biotec 130-118-978
NG2	1/500	Rabbit IgG	ab129051
NG2-PE	1/200	Rabbit IgG	C06035P
PDGFRa	1/1000	Rabbit IgG	CST#3174
PDGFRa-APC	1/50	Mouse IgG1	BioLegend #323512
MBP	1/50	Rabbit IgG	CST#78896
GFAP	1/400	Mouse IgG	G-3893
Islet1	1/50	Rabbit IgG	ab20607
beta III Tubulin(TUJ1)	1/1000	Rabbit IgG	ab18207
hNA(HuNu)	1/200	Mouse IgG	MAB1281
NeuN	1/3200	Rabbit IgG	CST#24307
A2B5	1/500	Mouse IgM	Invitrogen#433110
SOX10	1/1000	Rabbit IgG	ab264405
p-OLIG2 (S147)	1/500	Rabbit IgG	Bioworld AP0734
CEPT1	1/500	Rabbit IgG	20496-1-AP
GAPDH	1/5000	Mouse IgG	ab8245
β-actin	1/2000	Rabbit IgG	ab8227

Table S3. Sequence of primers for qPCR analysis.

Gene name	Species	Sequence of promers	
β-actin	Human	Forward	CCAGAGCCCGATGACCTTTTT
		Reserve	CACTGCCTCCTAGCTTGTCC
Olig2	Human	Forward	GCCTCGTATGTGAGGCAAAA
		Reserve	TCATCAAGAAATGTCGCACG
CSPG4	Human	Forward	CTTTGACCCTGACTATGTTGGC
		Reserve	TGCAGGCGTCCAGAGTAGA
PDGFRa	Human	Forward	TGGCAGTACCCCATGTCTGAA
		Reserve	CCAAGACCGTCACAAAAAGGC
ST8SIA1	Human	Forward	GTCCTCTGTTGGCTCTACATCT
		Reserve	CCCCGTCATACCACATGCTC
SOX10	Human	Forward	CCTCACAGATCGCCTACACC
		Reserve	CATATAGGAGAAGGCCGAGTAGA
NKX2.2	Human	Forward	GAGGACGACGACGAATACAAC
		Reserve	GTTCGAGGGTTTGTGCTTCTT
PAX6	Human	Forward	TGGGCAGGTATTACGAGACTG
		Reserve	ACTCCCGCTTATACTGGGCTA
NESTIN	Human	Forward	CTGCTACCCTTGAGACACCTG
		Reserve	GGGCTCTGATCTCTGCATCTAC
NANOG	Human	Forward	TTTGTGGGCCTGAAGAAAACT
		Reserve	CCATCGGAGTTGCTCTCCA
POU5F1	Human	Forward	CTGGGTTGATCCTCGGACCT
		Reserve	CCATCGGAGTTGCTCTCCA
PLP1	Human	Forward	ACCTATGCCCTGACCGTTG
		Reserve	TGCTGGGGAAGGCAATAGACT
NGN2	Human	Forward	AGGAAGAGGACGTGTTAGTGC
		Reserve	GCAATCGTGTACCAGACCCAG
SOX9	Human	Forward	AGCGAACGCACATCAAGAC
		Reserve	CTGTAGGCGATCTGTTGGGG
HB9	Human	Forward	CTCCTACTCGTACCCGCAG
		Reserve	TTGAAGTCGGGCATCTTAGGC
SMARCA4	Human	Forward	GACCAGCACTCCCAAGGTTAC
		Reserve	CTGGCCCGGAAGACATCTG
CEPT1	Human	Forward	ATGTGGAGATTCTCACCCGGA
		Reserve	TCTTCTAGCCGCTTTAGTTGGT
CHPT1	Human	Forward	CACCGAAGAGGCACCATACTG
		Reserve	CCCTAAAGGGGAACAAGAGTTTG
PPARG	Human	Forward	GGGATCAGCTCCGTGGATCT
		Reserve	TGCACTTTGGTACTCTTGAAGTT
PPARD	Human	Forward	CAGGGCTGACTGCAAACGA
		Reserve	CTGCCACAATGTCTCGATGTC
PPARA	Human	Forward	ATGGTGGACACGGAAAGCC
		Reserve	CGATGGATTGCGAAATCTCTTGG
MBP	Human	Forward	GGCCGGACCCAAGATGAAAA
		Reserve	CCCCAGCTAAATCTGCTCAGG
MOG	Human	Forward	GGCAGCAATGGAATTGAAAGTAG
		Reserve	TGGGGTCCTAGAACACCAAAG
MAG	Human	Forward	GGTGTCTGGTACTTCAATAGCC
		Reserve	CTCTCGTGGACTACTTGGGTG
BDNF	Rat	Forward	GCTGCTGGATGAGGACCAGA
		Reserve	GCTGCTGGATGAGGACCAGA
β-actin	Rat	Forward	GCCTTCCTTCTTGGGTAT
		Reserve	GGCATAGAGGTCTTTACGG

Table S4. Sequences of shRNA assays.

RNAi Name	Species Specificity	Target sequences
CEPT1 shRNA1	Human	5`- ACTGTAGCAGGGACCATATTT-3`
CEPT1 shRNA2	Human	5`- GGCACCTCTGTGGGCATATAT-3`
CEPT1 shRNA3	Human	5`- TGGTAACACGCCCTAACTATC-3`
Scramble shRNA	Huamn	5′ -GATCTCGCTTGGGCGAGAGTAA -3′

Supplementary Figures

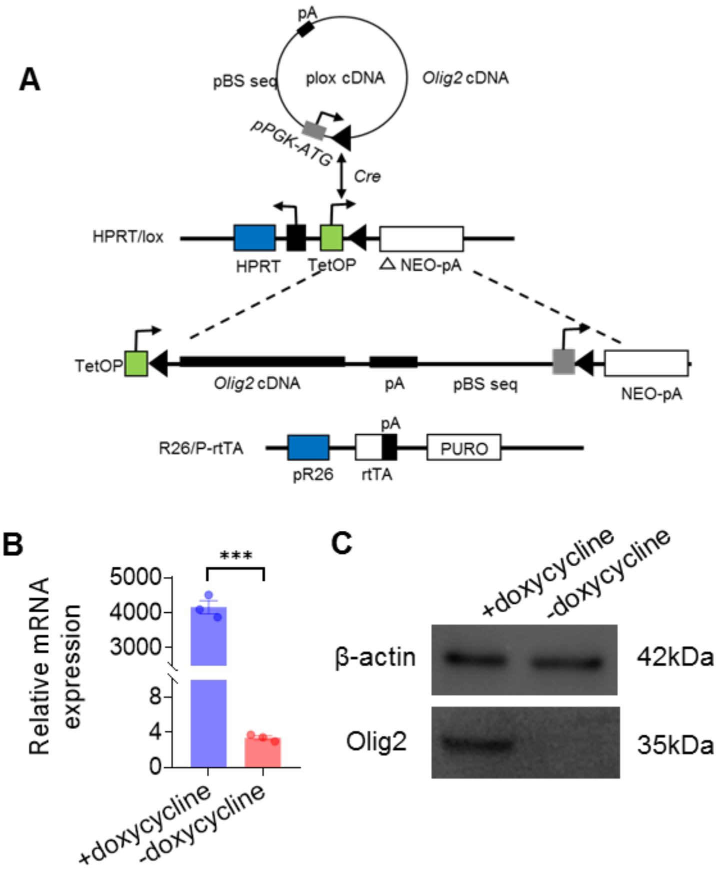

Figure S1 Establishment of hiPSC cell lines with induced expression of Olig2. **A** Schematic diagram of Tet-inducible Olig2 expression in hiPSCs. **B** qPCR analysis of Olig2 mRNA expression levels with treated or untreated doxycycline after 24 h (1 μg/mL) (n = 3, *** $p < 0.001$, by a two-tailed Student's t test). **C** Western blot for Olig2 expression with treated or untreated doxycycline after 24 h. mRNA and protein levels were normalized to the housekeeping gene β-actin. Graphs represent the individual data points and the mean ± SEM of three independent experiments.

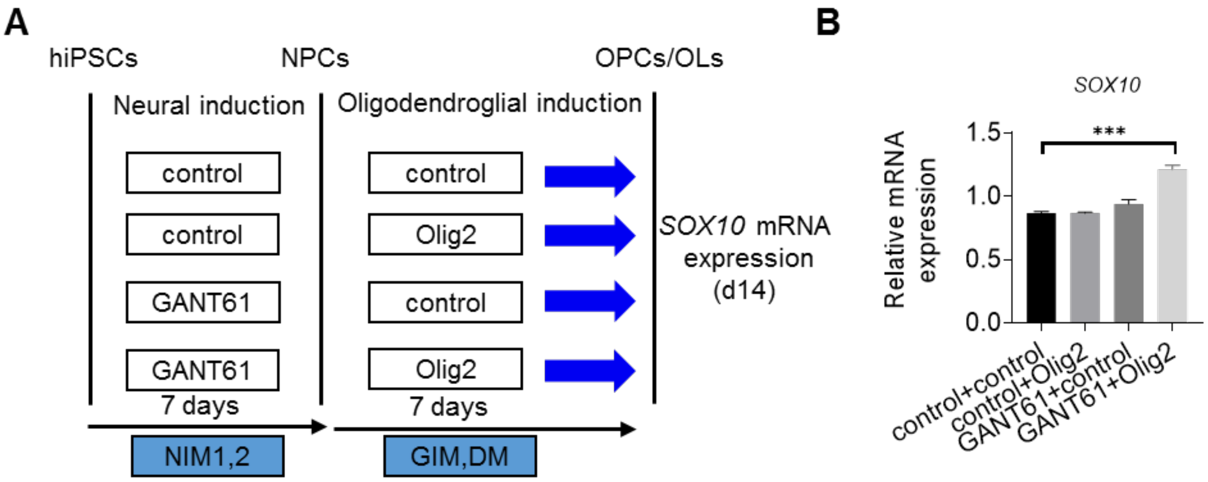

Figure S2 **A** Diagram of hiPSCs differentiated into NPCs and OPCs/OLs with untreated or treated GANT61, followed by induction of Olig2 expression. **B** qPCR analysis of *SOX10* mRNA expression at day 14 of differentiation (n = 3, *** p < 0.001). Graphs represent the individual data points and the mean ± SEM of three independent experiments.

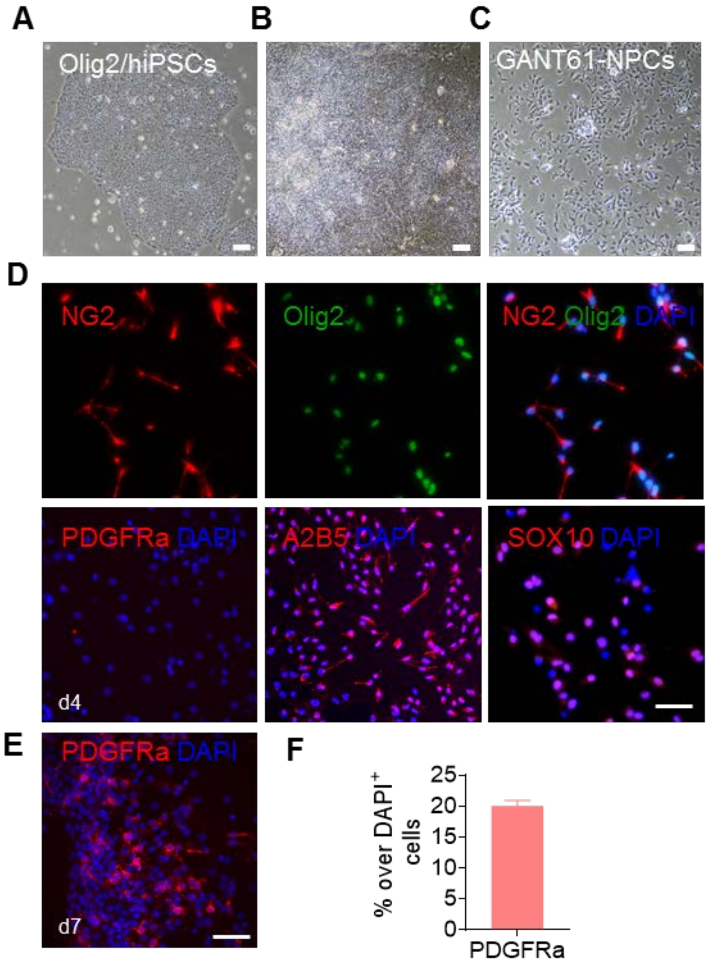

Figure S3 Adherent hiPSC (**A**) colonies treated with NIM1 for 2 days, followed by NIM2 for another 5 days, generated neural tube-like structures (**B**). The cultured cells were dissociated as single cells in six-well plates precoated with Matrigel. Confluent GANT61-NPCs (passage 2) were cultivated in adherent monoculture and maintained typical neural crest morphology during *in vitro* culture (**C**). **D** Representative immunofluorescent staining images of Olig2, NG2, SOX10, PDGFRa, and A2B5 in Olig2 cultures at d4 after Olig2 induction (scale bar, 50 μm). **E** Representative immunofluorescent staining images of PDGFRa in Olig2 cultures at d7 (scale bar, 100 μm).

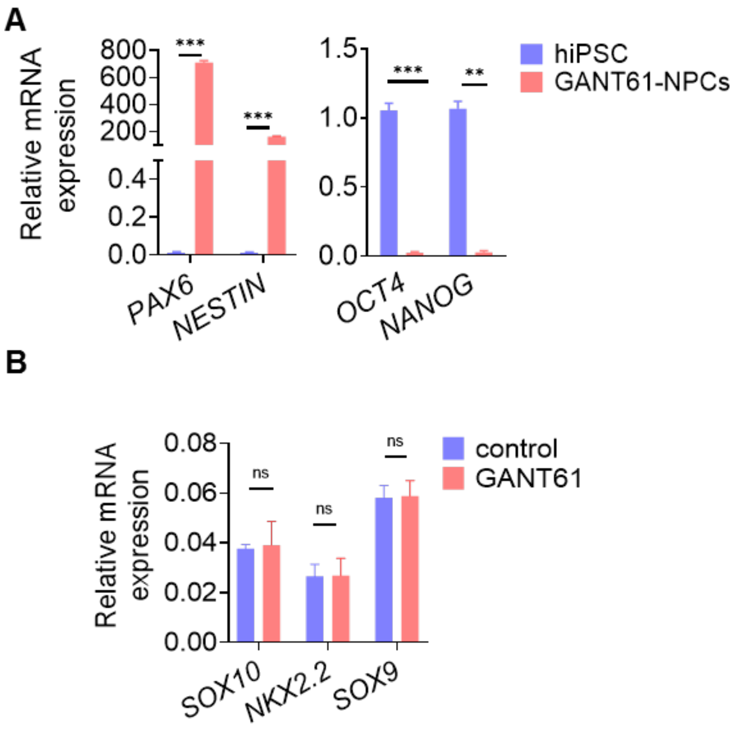

Figure S4 A The relative mRNA expression of GANT61-NPCs makers (*PAX6*, *NESTIN*) and pluripotency genes (*OCT4*, *NANOG*) in undifferentiated hiPSCs were detected by qPCR (n = 3, ** $p < 0.01$, *** $p < 0.001$, by a two-tailed Student's t test). B qPCR analysis for the OL-specific lineage maker genes *SOX10*, *NKX2.2* and *SOX9* mRNA expression level (ns, not significant, $p > 0.05$).

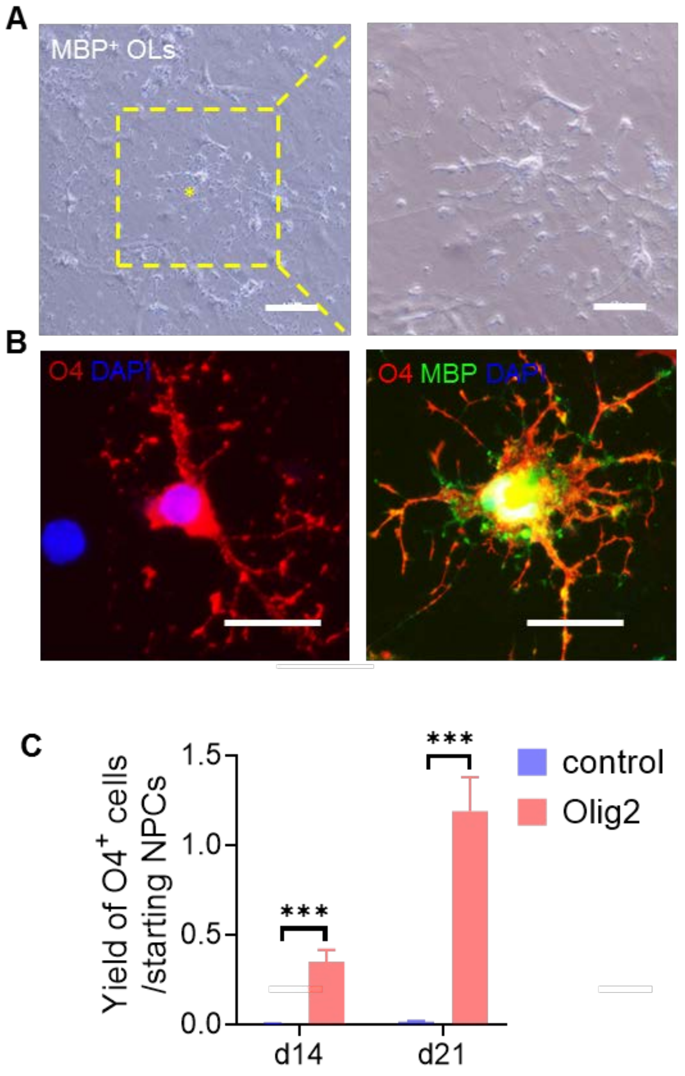

Figure S5 A Representative images for Olig2-induced OLs (scale bar, left, 100μm; right, 50 μm). **B** by d21, the Olig2-induced OLs coexpressed O4-epitope, the more mature OL marker, MBP (scale bars, 50 μm). **C** Quantification of O4^{+} mature OL yields at d14 and d28 after Olig2 transduction (n = 3, *** p < 0.001).

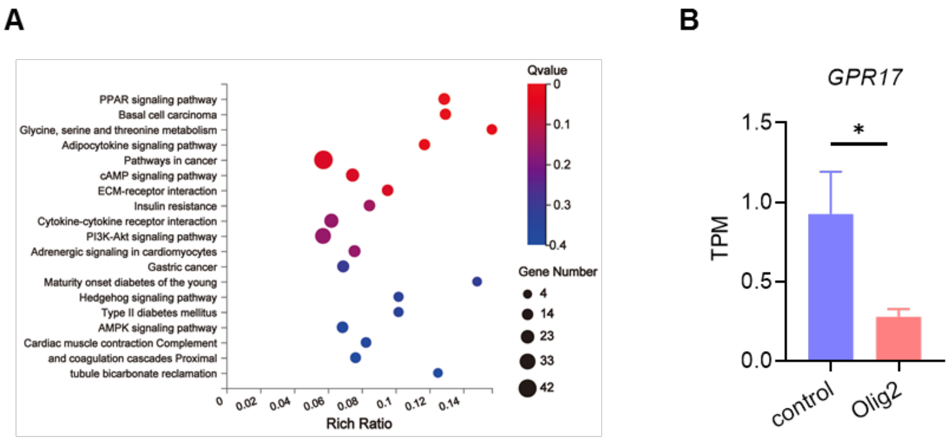

Figure S6. A KEGG pathway enrichment analysis of the Olig2/control-OPC-related mRNA-based RNA-seq data. The PPAR signaling pathway was highly enriched in Olig2-OPCs, and $p < 0.05$ was used as the threshold to select KEGG terms. **B** RNA-seq data indicated that the *GPR17* mRNA expression level was significantly downregulated in Olig2 OPCs ($n = 6$, * $p < 0.05$, by a two-tailed Student's t test).

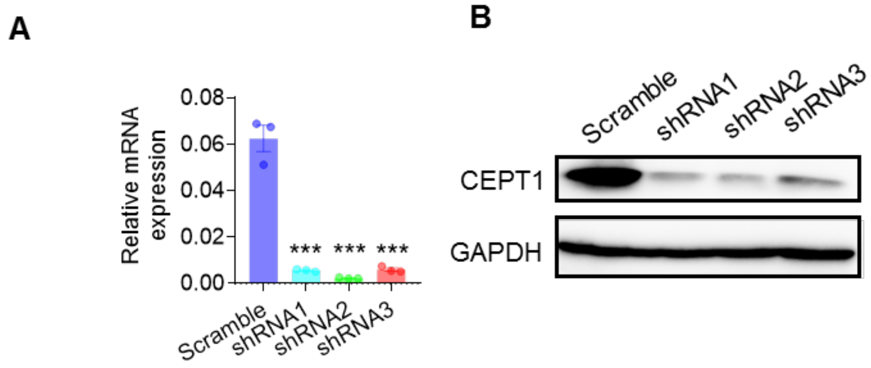

Figure S7 A qPCR analysis of *CEPT1* knockdown efficiency (n = 3, *** $p < 0.001$, by a two-tailed Student's *t* test). **B** Western blot analysis of CEPT1 knockdown efficiency. Proteins were normalized to GAPDH.

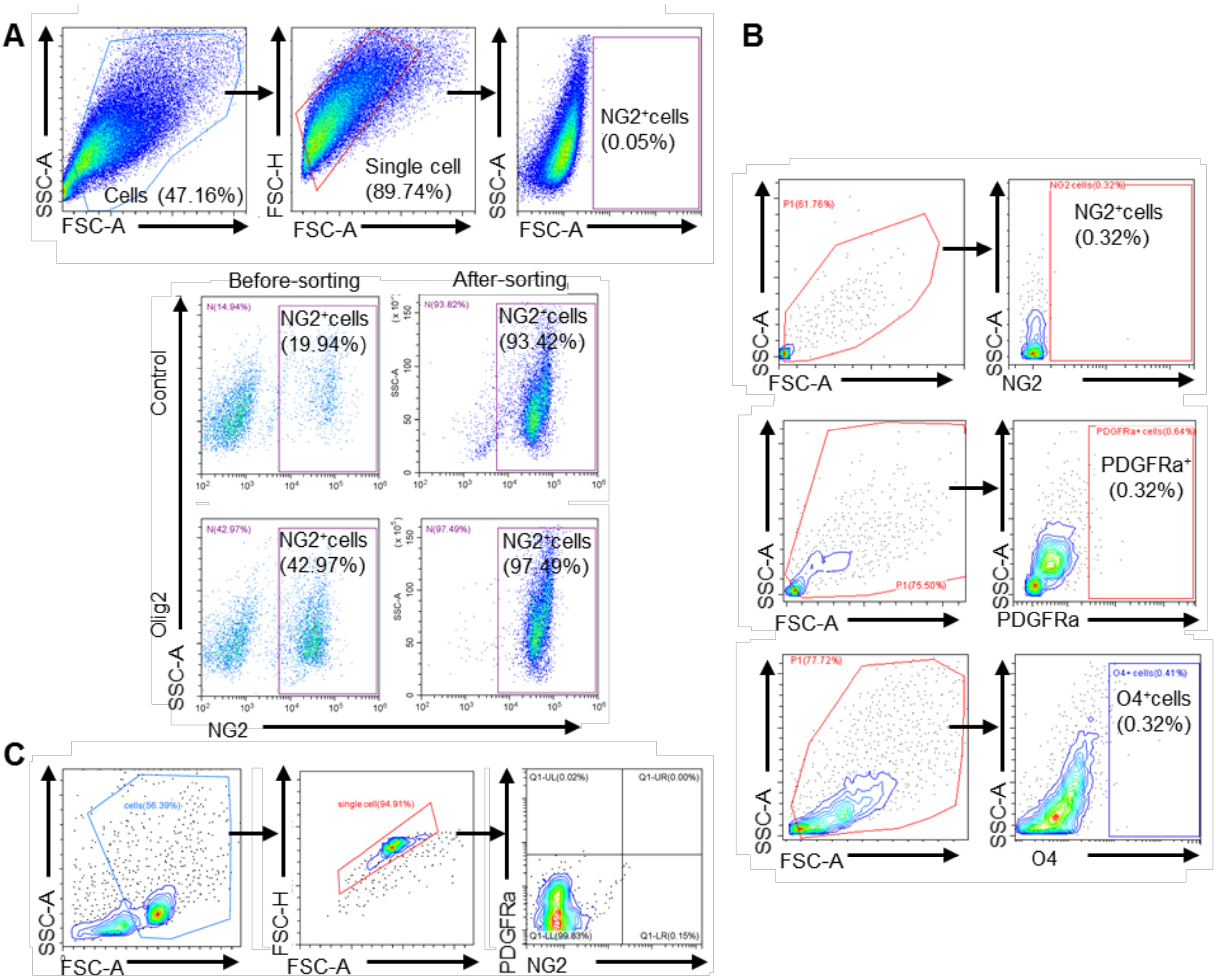

Figure S8 A Gating strategies to purify NG2^{+} OPCs for RNA-seq and cell transplantation. **B** Gating strategies to analyze the expression of the OPC-specific surface protein markers NG2, PDGFRa, and O4 in control-OPCs and Olig2-OPCs. **C** Gating strategies to analyze the coexpression of OPC-specific surface markers NG2 or PDGFRa in control-OPCs and Olig2-OPCs.

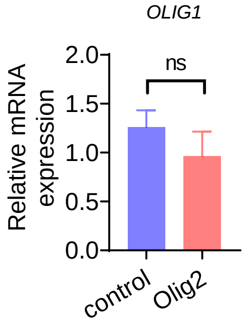

Figure S9 qPCR analysis for the mRNA expression level of *OLIG1* at 1 weeks after Olig2 induction. Olig2 overexpression did not affect the mRNA expression of *OLIG1* (n = 3, ns p > 0.05, by a two-tailed Student's t test).
